# Agglomeration Behavior and Fate of Food-Grade Titanium Dioxide in Human Gastrointestinal Digestion and in the Lysosomal Environment

**DOI:** 10.3390/nano13131908

**Published:** 2023-06-22

**Authors:** Francesca Ferraris, Andrea Raggi, Jessica Ponti, Dora Mehn, Douglas Gilliland, Sara Savini, Francesca Iacoponi, Federica Aureli, Luigi Calzolai, Francesco Cubadda

**Affiliations:** 1 Istituto Superiore di Sanità—National Institute of Health, 00161 Rome, Italy; francesca.ferraris@iss.it (F.F.); andrea.raggi@iss.it (A.R.); sara.savini7@gmail.com (S.S.); francesca.iacoponi@iss.it (F.I.); federica.aureli@iss.it (F.A.); 2 European Commission, Joint Research Centre (JRC), 21027 Ispra, Italy; jessica.ponti@ec.europa.eu (J.P.); dora.mehn@ec.europa.eu (D.M.); douglas.gilliland@ec.europa.eu (D.G.); luigi.calzolai@ec.europa.eu (L.C.)

**Keywords:** titanium dioxide, E 171, fate, human gastrointestinal digestion, lysosomes, particle agglomeration, physicochemical characterization, single-particle ICP-MS, transmission electron microscopy, risk assessment

## Abstract

In the present study, we addressed the knowledge gaps regarding the agglomeration behavior and fate of food-grade titanium dioxide (E 171) in human gastrointestinal digestion (GID). After thorough multi-technique physicochemical characterization including TEM, single-particle ICP-MS (spICP-MS), CLS, VSSA determination and ELS, the GI fate of E 171 was studied by applying the in vitro GID approach established for the regulatory risk assessment of nanomaterials in Europe, using a standardized international protocol. GI fate was investigated in fasted conditions, relevant to E 171 use in food supplements and medicines, and in fed conditions, with both a model food and E 171-containing food samples. TiO_2_ constituent particles were resistant to GI dissolution, and thus, their stability in lysosomal fluid was investigated. The biopersistence of the material in lysosomal fluid highlighted its potential for bioaccumulation. For characterizing the agglomeration degree in the small intestinal phase, spICP-MS represented an ideal analytical tool to overcome the limitations of earlier studies. We demonstrated that, after simulated GID, in the small intestine, E 171 (at concentrations reflecting human exposure) is present with a dispersion degree similar to that obtained when dispersing the material in water by means of high-energy sonication (i.e., ≥70% of particles <250 nm).

## 1. Introduction

Nanoparticles entering the human body via the oral route are subjected to conditions that are very different from those encountered via other exposure routes. Examples of such conditions are the extremely low pH of the stomach, which may promote the partial oxidation/dissolution of nanoparticles constituted of soluble metals/metal oxides with the release of constituent ions [1,2], or the high ionic strength in the stomach and intestine, which may lead to particle agglomeration in the gastric phase and, due to concurrent changes in pH and other factors, to deagglomeration in the intestinal phase [1,3,4].

Though the fate of nanomaterials during human digestion is still poorly understood, the results of particle interactions with the digestive milieu appear to be driven by several factors, including the physicochemical properties of the particles (e.g., size, shape, charge and surface properties), the nature of any co-ingested substance, the gastrointestinal (GI) environment and host factors, e.g., variability in the gastric pH (associated with lifestage and inter-individual differences) or in GI transit time [5]. Another aspect to be considered is that nanomaterials readily adsorb proteins and other biomolecules present in food and in the gut environment and the resulting corona, on the one hand, affects particle dispersion, and on the other, influences the biological behavior of the particles [6].

The available evidence indicates that the interactions with the GI environment may substantially alter the physicochemical properties of ingested particulate materials [1,2]. Changes in the size-related properties, shape or surface characteristics may affect the intestinal uptake of the particles and their toxicological properties [7].

New approach methodologies (NAMs) have enormous potential to improve the mechanistic understanding of toxicokinetic and toxicodynamic processes at the nanoscale by focusing on human-relevant models and avoiding animal testing. In the EU framework for the safety assessment of nanomaterials in food-related applications, nanoscale specificities are integrated in the risk assessment process as nanoscale-based hypotheses, and NAMs are the first choice to generate information for addressing these hypotheses [8]. Integrated Approaches to Testing and Assessment (IATAs) are then used for the integration of human, animal and NAM-derived evidence. Specifically, the guidance of the European Food Safety Authority (EFSA) on the risk assessment of nanomaterials to ensure consumer protection prescribes, at lower tiers, the use of in chemico methods for fate testing under GI and lysosomal conditions [9]. For GI processes, the focus is on the small intestine (do particles dissolve or do they reach the small intestine as such, with potential uptake and crossing of the intestinal barrier?), and a quantitative approach is required (determination of the dissolution rate profile by measuring the mass fraction of the original material present as particles after 30 min of in vitro simulated intestinal digestion). A similar quantitative approach is proposed for testing the biopersistence of the particles that do not dissolve in the GI tract (GIT) and may be taken up systemically (determination of the dissolution rate profile at 72 h in simulated lysosomal conditions to assess any potential for accumulation). The focus is on particles up to approximately 250 nm since available evidence suggests that specific cellular uptake mechanisms exist for particles within such a size range.

Food-grade titanium dioxide (also known as E 171) is a widely used food additive which, owing to the light-scattering effect of TiO_2_ particles occurring in the particle size range of 200–300 nm, lends a bright and natural white color [10]. It is also used as an excipient in oral medicines. The fraction of constituent particles with a minimum external dimension <100 nm in E 171, assessed via quantitative transmission electron microscopy (TEM) analysis, has been found to lie in the range 18–82% by number and 2–41% by mass; in addition, the same studies showed a negligible proportion of particles smaller than 30 nm to be present [11,12]. In 2021, the EFSA completed a re-assessment of E 171 safety as a food additive based on the available body of evidence concerning TiO_2_ nanomaterials with constituent particles >30 nm and food-grade titanium dioxide [13]. In this assessment, along with a potential for accumulation, immunotoxicity, inflammation, the induction of aberrant crypt foci (by E 171) and neurotoxicity (by nanoparticulate forms), TiO_2_ particles were found to possibly induce DNA strand breaks and chromosomal damage, with no certainty about a threshold mode of action. The concern for genotoxicity and the many uncertainties led to the conclusion that E 171 could no longer be considered safe, which led to regulatory actions resulting in the ban of E 171 as a food additive in the EU.

Following the EFSA’s scientific opinion, other bodies have recently assessed the risk of titania as food additive [14,15,16]. These assessments, carried out via a non-nano specific approach—i.e., without a structured conceptual framework to take into account nanoscale considerations [8]—concluded that there is currently no evidence to suggest that dietary exposure to food-grade titanium dioxide is a concern for human health. One main assumption behind these assessments was that E 171 particles in food are largely agglomerated and that the relevance of the toxicity studies in which food-grade titania was accurately dispersed in water via sonication and administered via oral gavage or via drinking water is very limited. A second assumption was that there is no evidence that TiO_2_ in foodstuffs may become more dispersed in the GIT, where increasing agglomeration was anticipated instead. This second assumption was based on the results of several studies [17,18,19,20,21,22,23], all conducted in fasted conditions or in the presence of simple components (e.g., caseins) but in the absence of an actual food matrix, in which either E 171 or non-food-grade TiO_2_ nanomaterials were placed in contact with simulated digestive fluids (mainly enzymes and electrolytes) at specific pHs to simulate human GIT processes. In these studies, light-scattering techniques, such as dynamic light scattering (DLS) and laser diffraction, were used to obtain information about the size of the TiO_2_ particles in suspension. However, these techniques are not suited for screening the presence of small particles or characterizing the particle size distributions (PSDs) of polydisperse materials and, as such, are not accepted for regulatory safety assessment in the EU [8]. Such analytical limitations, which prevent the use of these techniques for measuring the particle size of pristine materials, are even more prominent when it comes to assessing particle size changes in complex suspensions where the TiO_2_ particles coexist with proteins (digestive enzymes) and other particulate agents that introduce further bias in light-scattering measurements. Chemically-specific analytical techniques selectively targeting TiO_2_ particles are necessary to obtain meaningful information on particle size and agglomeration degree in studies simulating human GIT processes.

In the present study, we addressed the knowledge gaps and the uncertainties regarding the agglomeration behavior and fate of food-grade titanium dioxide in human gastrointestinal digestion (GID). A representative sample of E 171 was submitted to a thorough physicochemical characterization using a state-of-the-art multi-technique approach. TEM was used to characterize the size and shape of the constituent particles and determine the number-based PSD. Single-particle ICP-MS (spICP-MS), a powerful analytical tool that provides particle- and elemental-specific data [24,25,26,27,28,29], was used to investigate the agglomeration state in liquid suspensions, and µs dwell times were employed to improve the analytical figures of merit. This technique can accurately measure the agglomeration state since it detects discrete particles in suspensions irrespective of their being ’primary’ (i.e., individual constituent particles) or agglomerates (i.e., ’secondary particles’ formed through the clustering of two or more constituent particles). PSDs derived from spICP-MS measurements were obtained using the isotope combination approach, whereby both ^47^Ti and ^48^Ti are monitored and the data integrated in such a way as to achieve number- based PSDs covering a wider size range, i.e., avoiding the underestimation of the fraction of larger agglomerates caused by considering ^48^Ti alone, a common approach used in the vast majority of studies [30]. Further characterization of the material was achieved via centrifugal liquid sedimentation (CLS), volume-specific surface area (VSSA) determination via the BET method, zeta potential and isoelectric point (IEP) determination via electrophoretic light scattering (ELS), whereas DLS was used to enable comparisons with data from other studies.

The GIT fate of this typical E 171 material was studied by applying the in vitro GID approach laid down in the EFSA guidance on risk assessment of nanomaterials [8]. As prescribed by the approach, the study was performed with three different concentrations, with the middle one representing the average estimated human exposure. A standardized protocol developed by the international INFOGEST network was used for the in vitro simulation of GID in order to allow for the replication of experiments and ensure inter-comparability of the results [31]. E 171 GIT’s fate was studied in fed conditions, and a cereal-based food, ensuring the concurrent presence of major dietary components (carbohydrates, protein, fat and fiber), was selected as model food. The experiment was repeated in fasted conditions, which are meant to be more predictive of E 171 behavior when used in food supplements and in oral medicines. In addition, with a view to complementing the results obtained in fed conditions, actual E 171-containing food samples were submitted to simulated digestion and the fate of TiO_2_ particles characterized.

A thorough check of TiO_2_ mass balance along the three-phase GIT cascade (oral, gastric and small intestinal) was carried out via total Ti determination using ICP-MS after microwave (MW) digestion, whereas potential dissolution was assessed via ultrafiltration followed by total Ti determination using ICP-MS. For characterizing the agglomeration degree in the small intestinal phase, spICP-MS represented an ideal analytical tool to overcome the limitations of earlier studies based on light-scattering techniques.

The fate of E 171 in lysosomal conditions was also studied to obtain data on the biopersistence of the material and its potential for bioaccumulation. Stability in lysosomal fluid was investigated by applying the in vitro approach laid down in the EFSA guidance on the risk assessment of nanomaterials [8] after close scrutiny of the existing protocols [32,33,34,35,36] and the selection of physiologically relevant conditions.

## 2. Materials and Methods

### 2.1. Instrumentation

A Nexion 350D ICP-MS (Perkin Elmer, Shelton, CT, USA) system equipped with a Meinhard concentric nebulizer, a glass cyclonic spray chamber and a standard quartz torch (2.5 mm i.d) was used for TiO_2_ characterization and quantification with the Syngistix™ Nano Application software v. 2.5 (Perkin Elmer, Shelton, CT, USA). Total titanium analyses were performed by means of an 8800 ‘Triple quad’ ICP mass spectrometer (Agilent Technologies Inc., Tokyo, Japan). A JEM 2100 TEM from JEOL (Milan, Italy), coupled with energy-dispersive x-ray spectroscopy (EDX, Brüker, Milan, Italy), was used for the characterization of constituent particles. VSSA determination via the BET method was carried out by means of a Micromeritics Gemini VII instrument (Micromeritics Instrument Corporation, GA, USA). Zeta potential and IEP were determined via ELS using a Malvern Nano ZS instrument (Malvern, UK); the same instrument was used for DLS determinations. CLS determinations were carried out using a DC24000 UHR instrument (CPS Instruments, Inc., Prairieville, LA, USA).

For the dissolution of TiO_2_ particles and determination of total Ti, samples were digested in an UltraWave Single Reaction Chamber Microwave Digestion System (Milestone, Bergamo, Italy). In vitro digestion was performed in a GFL 1083 temperature-controlled incubation chamber (GFL, Burgwedel, Germany). A Vial Tweeter ultrasonic device UP200St was used for particle dispersion (Hielscher, Teltow, Germany). Other pieces of equipment used were a Bandelin Sonorex RK 510 H ultrasonic water-bath and a Thermo Megafuge 11R centrifuge.

### 2.2. Reagents and Materials

Ultrapure water was obtained from a Milli-Q Element purification system with 0.22 µm filters (Millipore, Molsheim, France). HNO_3_ 67–69% *v/v* (ultrapure grade, Carlo Erba, Rodano, Italy), HF (ultrapure grade, Carlo Erba, Rodano, Italy) and H_2_O_2_ 30% *v/v* (ultrapure grade, Sigma-Aldrich, Darmstadt, Germany) were used for microwave digestions. Analytical-grade EtOH, DMSO and cyclohexane from Merck were used.

For spICP-MS measurements, the dissolved ionic gold standard (1 g L^−1^ in 5% HCl) and gold nanoparticles with a nominal diameter of 60 nm (43.45 μg mL^−1^ in aqueous 2 mM sodium citrate) were purchased from High-Purity Standards (Charleston, SC, USA) and NanoComposix (San Diego, CA, USA), respectively. Dissolved ionic titanium standard for ICP-MS (1 g L^−1^ in 2% HNO_3_) was purchased from High-Purity Standards (Charleston, SC, USA).

Pepsin from porcine gastric mucosa, pancreatin from porcine pancreas, and bile salts were obtained from Sigma-Aldrich. Gastric Lipase was obtained from Lypolitech (Marseille, France). All salts were purchased from Merck (Darmstadt, Germany).

Syringe filters of 5 µm (Sartorius, Goettingen, Germany) and Amicon^®^ Ultra-4 Centrifugal Filter Units (Ultracel-50 regenerated cellulose membrane, 4 mL sample volume) were obtained from Millipore (Molsheim, France).

### 2.3. E 171 Samples

E 171-a, a typical food-grade anatase sample, and E 171-b, i.e., food-grade anatase incorporated (15%) into a matrix consisting of hydroxypropyl methylcellulose (65%), microcrystalline cellulose (10%) and stearic acid (10%), were both obtained from FBOs. E 171-c (Titanium (IV) oxide, Emprove Essential, Ph. Eur., BP, ChP, JP and USP) was obtained from Merck. E 171-a and E 171-b were submitted to full characterization, and E 171-a was used as ‘model’ E 171 for investigating the agglomeration degree in fed and fasted GIT conditions. E 171-b and E 171-c were only submitted to dissolution studies.

### 2.4. Model Food and E 171-Containing Food Samples

Rusks (fat: 5%; saturated fat: 1%; carbohydrates: 77%; sugars: 5%; fiber: 3%; protein: 11%; salt: 2%) were chosen as the model food. The item was purchased from a local supermarket and homogenized by means of a Buchi B-400 with ceramic blades. The E 171-containing food samples, purchased from local markets, were (i) button-shaped chocolate candies and (ii) high-protein cappuccino, in which we determined TiO_2_ concentrations of 150 and 535 µg g^−1^ (fresh weight), respectively.

### 2.5. E 171 Multi-Technique Characterization

Sample treatment before characterization via the techniques listed hereunder is shown in Figure S1. Vial tweeter (indirect) sonication, instead of probe sonication, was used for dispersing the pristine materials. Even though the energy output is lower with indirect sonication, it avoids contamination via the release of Ti-containing particles from the probe tip, which may occur with direct sonication. The delivered energy for sonication via both vial tweeter and ultrasonic bath (used for other steps of the experimental protocols) was determined as reported in the Supplementary Material (SM).

#### 2.5.1. TEM

TEM analysis at 120 kV was used for the characterization of the constituent particles of pristine E 171 samples in terms of shape, minimum and maximum Feret diameter (Fmin and Fmax, i.e., width and length) and aspect ratio (Fmax/Fmin). A total of 3 µL of each dispersion was dried overnight on a 200 mesh Cu-formvar carbon-coated grid, and then, placed under high vacuum (10^−3^ Pa) to allow for complete water evaporation. The bright field signal was used for image formation, and image analysis was performed using ImageJ software version 20. The mean Feret diameter (Fmean) of each particle was calculated as the arithmetic average of Fmin and Fmax.

TEM with EDX was used in STEM mode, to characterize the titanium particles in the samples submitted to simulated digestion in fed conditions. A sample aliquot was filtered through glass filters (pore size: 2 µm) conditioned with MilliQ water in order to remove the undigested food residues. The filtrate, placed in a 50 mL falcon, was centrifuged for 10 min at 4200 rpm (3392 g). The formation of three phases was observed: the supernatant and the micellar part were removed (the first by decanting, the second using a spatula) and stored at −20 °C for subsequent analysis. In order to be analyzed via TEM, the pellet was purified and concentrated as detailed in Figure S2. The resulting dispersions were placed on TEM grids and treated as detailed above.

#### 2.5.2. VSSA

The Brunauer–Emmett–Teller (BET) method was used to determine the VSSA of E 171-a according to ISO 9277. The material was outgassed under vacuum at 200 °C for 24 h prior to measurements.

#### 2.5.3. DLS and ELS

Batch mode DLS measurements were performed using an instrument equipped with a 633 nm HeNe laser. The results were generated by averaging 10 consecutive measurements of 3 runs. A refractive index value of 2.49, an absorption of 0.3 and a viscosity (cP) pf 1.33 were considered. In order to assess the effect pH variations in the GIT environment, E 171 suspensions were prepared in deionized water (pH ca. 5.5), at salivary/small intestinal pH 7 (phosphate buffer 10 mM) and at gastric pH 2.89 (citric acid/sodium citrate buffer). Zeta potential and IEP determination were carried out via ELS using the same apparatus.

#### 2.5.4. CLS

CLS is based on the separation of particles from a liquid under the presence of a centrifugal force, with the settling velocity depending on particle characteristics such as size, density and shape. Measurements were performed in a CPS disc centrifuge equipped with a 405 nm laser, at 22,000 rpm, using an 8–24% (*w*/*w*) sucrose gradient. An aliquot (100 mL) of a 100-fold diluted particle suspension was injected into the centrifuge disk after calibration with 239 nm PVC particles. A refractive index value of 2.49, absorption of 0.075 and density of 3.84 g mL^−1^ were used as input parameters for the calculations.

### 2.6. Single-Particle ICP-MS

#### 2.6.1. Instrumental Analysis

The operating conditions were optimized daily to achieve maximum sensitivity. For setting all parameters and data acquisition, the Nano Application Module of the Syngistix™ (2.5) software was used. The dwell time was 100 μs and the total data acquisition time 60 s. The transport efficiency (TE) was determined daily following the ‘particle size approach’ [37]. The exact flow rate of the peristaltic pump required for TE determination was measured daily and was around 0.3 mL min^−1^ on average. TE was calculated daily, and the average value is shown in Table 1 along with the other instrumental parameters.

For the determination of the equivalent spherical diameter (ESD) of TiO_2_ particles, obtained from the particle mass assuming a spherical geometry, the Ti isotopes at m/z 48 and 47 were monitored. A 4-point calibration curve ranging from 0 to 30 μg L^−1^ dissolved titanium was used. Immediately before analysis, samples were diluted with ultrapure water to achieve a particle concentration of 1000–2000 particles per 60 s scan time (Table 1). The performance characteristics of the method are summarized in the Supplementary Materials.

#### 2.6.2. E 171 Dispersion Optimization for sp-ICP-MS Analysis

E171-a dispersion using plain MilliQ water and NaOH 0.1 mM pH = 10 [11] was compared. Two concentrations (0.1 mg/mL and 0.7 mg/mL) were tested.

### 2.7. Fate Studies

#### 2.7.1. Simulated Gastrointestinal Digestion

Sample handling was carried out in clean room conditions. Simulated salivary fluid (SSF), simulated gastric fluid (SGF) and simulated intestinal fluid (SIF) were prepared according to [31], as shown in Table S1. All the enzymes and bile salts were freshly prepared.

TEM analysis in fed conditions was performed on E 171-a (Figure S2) and, in order to confirm the chemical composition of the particles analyzed, EDX maps were acquired in STEM mode. Digestion in fed conditions was applied to E 171-a, the cappuccino (reconstituted as indicated on the product label) and the chocolate candies (treated as detailed in Figure S2). Fasted conditions were applied to E 171-a, E 171-b and E 171-c. For each sample, the experiment was performed in triplicate plus a procedural blank.

As per EFSA guidance [9], samples (1 mL) were taken during the intestinal phase at multiple time points (0; 5; 10; 15; 30; 60 min) for analytical measurements. A detailed study for optimizing sample handling in fed conditions was carried out by comparing centrifugation (4 mL, 1500 rpm, 5 min, 0 °C) and filtration for the removal of digestion residues. The experimental procedure shown in Figure S2 was selected on the basis of superior TiO_2_ mass recovery and also applied in fasted conditions for consistency. Each E 171 material was analyzed in triplicate with one procedural blank.

Test concentrations were selected on the basis of human exposure. From dietary intake estimates [38,39,40,41], two scenarios (lower and higher exposure) were drawn, i.e., 0.1 and 1 mg/kg bw per day, whereas 5 mg/kg bw per day was considered the worst-case scenario. The resulting concentrations were calculated as outlined in the EFSA guidance, considering 4 L as the daily volume of GIT secretions [9]; the dilution factor related to the procedure (×8) was applied and, after rounding, test concentrations of 0.01, 0.1 and 0.7 mg/mL were obtained.

#### 2.7.2. Lysosomal Digestion

The lysosomal simulated fluid (LSF) was prepared according to Stopford et al. [36] as shown in Table S2. For each E 171 sample, the experiment was performed in triplicate and a procedural blank was included, as reported in Figure S2.

The test concentration was 0.1 mg/mL. As per EFSA guidance [9] samples were taken at multiple time points (0; 2; 5; 24; 72 h) for analytical measurements.

Due to the long duration, the effect of carrying out the procedure in different sample containers was studied by comparing Falcon polypropylene tubes and glass-decontaminated tubes (Figure S3; Table S3).

#### 2.7.3. ICP-MS Analytical Measurements

Microwave digestion was performed in order to measure the total titanium present in the samples collected in Section 2.7.1 and Section 2.7.2, as detailed in the Supplementary Materials. To assess the presence of any dissolved fraction, total Ti after ultrafiltration was measured. Ultrafiltration through Amicon 50 KDa tubes (nominal size cut-off of 5 nm) was performed as detailed in the Supplementary Materials. Total titanium analysis via ICP-MS is described in the Supplementary Materials.

### 2.8. Statistical Analysis

PSDs are represented as histograms (bin size = 10 nm) with percentage frequencies and overlaid Kernel density functions. The PSD descriptors included measures of central tendency, percentiles and the percentage of particle diameters (in number or mass) below given size thresholds. All analyses were performed using SPSS v.28 (IBM SPSS Statistics).

## 3. Results

### 3.1. Pristine Material Characterization

#### 3.1.1. Characterization of the Constituent Particles via TEM and VSSA

**Figure 1:**
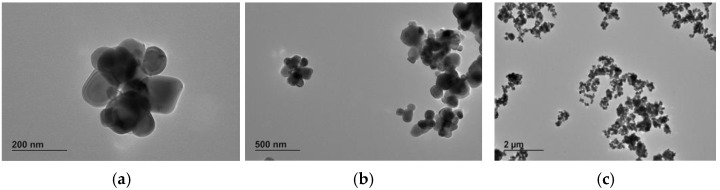
TEM images of pristine 171-a: (**a**) 30,000× magnification; (**b**) 10,000× magnification; (**c**) 2500× magnification.

**Figure 2:**
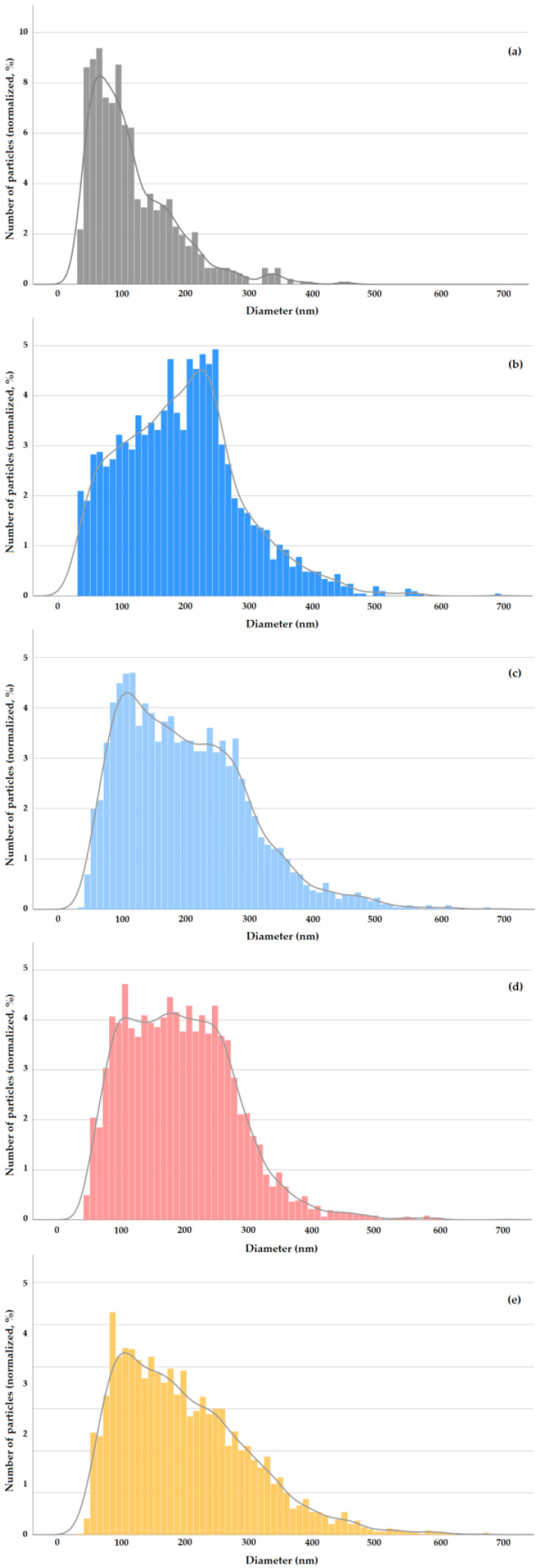
E 171 number-based PSDs. TEM (Fmean): E 171-a (**a**). spICP-MS (ESD): E 171-a (0.1 mg/mL) in MQW after sonication (**b**), E 171-a (0.1 mg/mL) after fasted GID (**c**), E 171-a (0.1 mg/mL) after fed GID (**d**) and E 171 in chocolate candies (0.15 mg/mL) after GID (**e**). For all GID data, the time point at 30 min of the intestinal phase is shown.

E 171-a exhibited the typical features of food-grade anatase [12], with compact structured constituent particles of mixed morphology including spheres and particles with irregular geometry consisting of polyhedrons (i.e., with a strong angular component) (Figure 1). Although the constituent nanoparticles have a prevalent Fmin diameter around 80 nm, smaller constituent nanoparticles with Fmin between 30 and 60 nm and larger particles with Fmin up to ca. 250 nm can be observed. The descriptors of the number-based PSD of E 171-a are summarized in Table 2, whereas the Fmean distribution is shown in Figure 2a. The VSSA of this material is 37 m^2^/cm^3^.

In contrast to this material, E 171-b was considered in the present study as an interesting case of an unusual formulation with other food-grade substances, resulting in the formation of an organic coating on the particle surface, clearly visible in the TEM micrographs (Figure S4); a detailed description of this material can be found in the Supplementary Materials.

#### 3.1.2. Multi-Technique Characterization of E 171 Water Suspensions

For the characterization of E 171 water suspensions and investigation of the agglomeration degree of particles, spICP-MS is an ideal technique. However, as shown in Figure 2a, food-grade TiO_2_ is a highly polydisperse material, with Fmean values spanning a range of 30–300 nm and a few constituent particles having Fmean diameters around 400 nm. This polydispersity makes it impossible to establish a complete PSD using a single isotope in spICP-MS. ^48^Ti, which is the most sensitive isotope—i.e., the one providing the lowest background equivalent diameters (BED, instrumental particle size detection limit)—for this same reason is prone to detector saturation when large TiO_2_ particles are atomized [26,27,28]. ^47^Ti and ^49^Ti are less abundant isotopes (7.44% and 5.41%, respectively) and provide BEDs increased by a factor of 2.15 to 2.39, respectively [29]. However, for this same reason, they enable larger TiO_2_ particles to be measured without falling outside the detector linear range due to saturation. Based on the fact that ^48^Ti gives access to smaller particles, while ^47^Ti and ^49^Ti give access to larger particles [30], in this study, both ^48^Ti and ^47^Ti were measured and the data combined (see Supplementary Materials). This allowed us not to lose the larger particles and to obtain more accurate number-based PSDs (i.e., more faithful representations of actual size ranges), which led to improved mass balances (Table S6).

With this approach, the ESD number distributions summarized in Table 3 were obtained for E 171-a (further details in Table S7). Since the concentration is the most influential parameter affecting particle agglomeration in suspension, two concentrations (i.e., 0.1 and 0.7 mg TiO_2_/mL) were tested. Dispersion in plain MilliQ water was compared with dispersion at pH = 10 in NaOH 0.1 mM [11]. The results were similar (Table 3 and Table S7), and thus, plain water dispersion was adopted for subsequent studies. The PSD of E 171-a in water at 0.1 mg TiO_2_/mL is shown in Figure 2b.

The mass-based arithmetic mean diameter determined from PSDs obtained via CLS for E 171-a at 0.1 mg TiO_2_/mL was 233 nm (see Supplementary Materials for details and for E 171-b data). This value fits well with the mode of the PSD and the nearby cluster of high-frequency classes determined via spICP-MS at the same concentration (Figure 2b).

Even though DLS cannot provide meaningful PSD data for polydisperse materials, this technique has been used in a number of studies on E 171, and it is considered here to show its inherent limitations when compared to CLS or spICP-MS. The intensity-weighted mean hydrodynamic diameter of E 171-a at 0.1 mg TiO_2_/mL measured via DLS in plain water (pH 5.5) was 337 nm (Table 4) (see Supplementary Materials for details and for data referring to E 171-b). At intestinal and gastric pH (i.e., 7.0 and 2.9, respectively), the mean hydrodynamic sizes become 373 nm and 811 nm, respectively. In all cases, a small peak at ca. 5 µm is present, but considering that the scattering intensity is proportional to the sixth power of the particle diameter, this peak is negligible on a particle number basis. Zeta potential values (Table S9) show that at neutral pH, a more stable dispersion is obtained for E 171-a. Interestingly, E 171-b surface properties (including particle charge) are altered by the organic coating, and this material exhibits remarkably low stability when dispersed in water independently of the pH (Table S10).

### 3.2. Simulated Gastrointestinal Digestion

#### 3.2.1. Protocol Optimization and Fate Assessment

The fate of E 171 along the human GID was studied using the protocol outlined in Figure S2. At all time points of the intestinal phase, samples were taken to check, via ultrafiltration and total Ti analysis, whether any dissolution occurred. At the start of the intestinal phase and after 30 min, additional analyses were performed to check mass recovery (via total Ti analysis) and to assess the E 171 agglomeration state (via spICP-MS).

Protocol optimization entailed a study of the conditions to better achieve removal of the undigested food residues in fed GID conditions via either centrifugation or filtration; TiO_2_ mass recovery was five times higher with filtration on 5 µm filters (Table S11), and this procedure was adopted in both the fed and fasted protocols for consistency. Before filtration, 1:100 dilution was applied, which also helped reduce the salt content before total titanium analysis via ICP-MS.

TiO_2_ mass loss, potentially occurring during the various steps of the whole experimental procedure, was checked. Whereas 5 µm filtration in MQW was found to greatly affect TiO_2_ recovery (probably due to sticking of the particles to the filter surface), this was not confirmed in GIT conditions (especially with the fed GID) (Table S12), which highlights the importance of simulating the physiological environment (e.g., ionic strength and the presence of enzymes) in the optimization of experimental protocols.

E 171-a in fed conditions was analyzed via TEM after 30 min of intestinal simulated digestion, in order to assess the material’s persistence by looking at the shape and the size of the particles. The TEM images showed that titanium dioxide particles were not affected by the digestion process, with their size and shape being undistinguishable from those of the pristine material (Figure S17). To confirm the chemical nature of the particles, STEM maps were acquired (Figure S18).

For the three E 171 samples tested, both in fed and in fasted conditions, and irrespective of the tested concentrations, the measured dissolved Ti concentrations were found to be <LOD (0.13 µg/kg) at all time points, showing that TiO_2_ particles do not dissolve during human GI digestion.

#### 3.2.2. Agglomeration Behavior in the Intestinal Phase

The ESD number distributions obtained for E 171-a- and real E 171-containing samples submitted to GID, as determined via spICP-MS, are summarized in Table 5 (further details are given in Table S13). The PSDs at two time points of the intestinal phase, i.e., at the start and after 30 min, were characterised. The data for E 171-a refer to both fasted (i.e., in the absence of the model food) and fed (i.e., with the presence of the model food) conditions and to three different test concentrations, with the middle one (0.1 mg TiO_2_/mL) representing the average estimated human exposure to E 171. The number PSDs at this middle concentration (and at T = 30 min) are shown in Figure 2c,d for the fasted and fed digestion, respectively. Figure 2e shows the number PSD at T = 30 min for E 171 in chocolate candies.

The main PSD descriptors for E 171-a in fasted conditions at 0.1 mg TiO_2_/mL are very similar to those of E 171 in water at the same concentration (Table 3). The shape of the distribution changes somehow, since the cluster of high-frequency classes (220–260 nm in water, Figure 2b) moves to lower sizes after GID (90–120 nm, Figure 2c). However, the median ESD values, as well as the proportion of particles smaller than 100 nm and 250 nm, are almost identical (Table 3). This is remarkable, since E 171 in water was sonicated to improve deagglomeration and dispersion stability, whereas E 171 after GID was analyzed as is (no energy delivered via sonication). Moving to fed conditions, it emerges that the presence of an actual food matrix leads to an improvement in particle deagglomeration, as can be seen by comparing the median ESD and the proportion of particles <250 nm between each corresponding experimental point obtained in fasted conditions at the three different concentrations tested (Table 3). This is also confirmed by the comparison in Figure 2d,c, where it is apparent that in fed conditions, the right-hand-side tail of the distribution is reduced (i.e., fewer particles larger than 250 nm are present). The effective particle dispersion caused by the presence of a food matrix is further confirmed by the PSDs obtained for chocolate candies submitted to GID (Table 5, Figure 2e), notwithstanding the higher E 171 concentration present in this case (i.e., 0.15 mg/mL).

**Figure 3:**
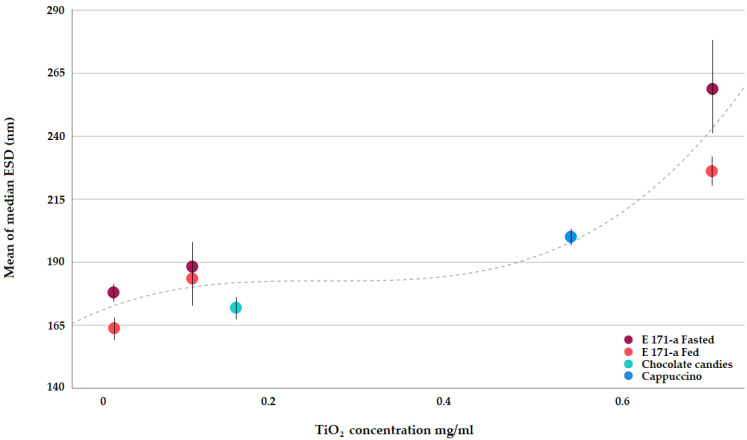
Median E 171 ESDs after simulated GI as determined via spICP-MS.

Figure 3 clearly shows that, when submitted to the simulated human GID, E 171 presents a quite uniform degree of dispersion. Slightly improved deagglomeration is achieved in the presence of a food matrix (see fasted vs. fed conditions at the same concentration), but overall, TiO_2_ in the intestinal phase of human digestion remains fairly well dispersed over a wide concentration range, reflecting the range expected as a result of human oral exposure.

Only at the highest concentration tested (0.7 mg/mL), does a trend towards greater agglomeration become visible (Figure 3). It is noted that such a concentration corresponds to worst-case human exposure conditions, and still, in such a scenario, 61% of the particles present in the small intestinal phase are smaller than 250 nm (Table 5), i.e., readily available for absorption by the intestinal epithelia via particle-specific uptake mechanisms.

### 3.3. Lysosomal Digestion

Optimization of the lysosomal protocol entailed an assessment of the material minimizing TiO_2_ adsorption on the container walls during the 72 h long process (Figure S3 and Table S3). Under the optimized conditions, the measured dissolved Ti concentrations were < LOD (0.13 µg/kg) at all time points, showing that TiO_2_ particles do not dissolve in lysosomal conditions.

## 4. Discussion

In the present study, the GIT fate of E 171 was studied by applying the EFSA guidance on the risk assessment of nanomaterials [9] and using a standardized international protocol for the in vitro simulation of GID [31]. E 171 was confirmed to be resistant to dissolution, and thus, its stability in lysosomal fluid was investigated, again, in accordance with the above-mentioned guidance [9]. Biopersistence of the material after lysosomal processing was confirmed, which is in agreement with the long half-life estimated in vivo and the consequent, well-recognized tissue accumulation potential [13,42,43,44].

Using state-of-the-art analytical approaches, we demonstrated that, after simulated GID, in the small intestine, E 171 at the concentrations expected from average human exposure is present, with a dispersion degree similar to that obtained when dispersing the material in water by means of high-energy sonication. This is indeed in line with previous observations on the agglomeration state of E 171 present in food [45] and not unexpected on the basis of known factors affecting the dispersion of TiO_2_ particles in aqueous suspensions [46].

In general terms, particles are under the influence of attractive and repulsive forces, and agglomeration occurs when the attractive forces, which are higher as the particle size decreases, are predominant [47]. Constituent particles in agglomerates are held together by weak forces, such as van der Waals forces or simple physical entanglement. Contrary to aggregates, in which particles are strongly (i.e., non-reversibly) bound, agglomerates can be broken down depending on the strength of the external forces. When pristine E 171 is suspended in water, a considerable amount of energy from sonication is needed to overcome the strong attractive forces among the particles and counteract their agglomeration propensity [47]. In the small intestinal environment, and especially when E 171 is ingested along with food, factors such as (i) the smaller particle concentration (some of the particles are entrapped in the solid digesta and are slowly released), (ii) steric stabilization by enzymes, biomolecules and food components, and (iii) favorable pH (which is in the stability region further away from the IEP, i.e., the point at which the electrostatic repulsion is non-existent) yield stable dispersions [45,47].

When E 171 reaches the small intestinal compartment without the concomitant presence of food, i.e., in fasted conditions such those resulting from its ingestion, e.g., in tablets (food supplements and oral medicines), the results obtained in the present study show that the level of dispersion, albeit lower than that in fed conditions, is still substantially high (no less than 70% of the particles have a size <250 nm in the most likely concentration range, resulting from estimated human exposure).

The results of the present study are opposite to those obtained in a series of studies in which light-scattering techniques, such as DLS and laser diffraction, were inappropriately used to obtain information they cannot deliver, i.e., data on titania agglomeration in GID [17,18,19,20,21,22,23]. Whereas DLS can provide some size-related information for pure monodisperse nanomaterials [48], it cannot be used for polydisperse materials and, as such, is not an accepted technique for regulatory safety assessment in the EU [8]. We showed herein that DLS produces biased results for E 171 dispersed in water, and we consider that its use in complex suspensions, where the TiO_2_ particles coexist with proteins (digestive enzymes) and other particulate agents with light-scattering properties, is analytically unjustifiable.

In some recent risk assessments of titania as a food additive, the above-mentioned data obtained using light-scattering techniques were used to support the assumption that E 171 extensively agglomerates in the human small intestine [14,15,16]. Another assumption of these assessments was that E 171 particles in food are largely agglomerated, and that the toxicological studies in which food-grade titanium was accurately dispersed in water via sonication and administered via oral gavage or via drinking water are of limited relevance [14,15,16]. The results of the present study show that this assumption is unsupported and evidence goes in the opposite direction. In addition, moving from these two assumptions, these recent reviews attributed the highest weight to toxicological studies in which food-grade TiO_2_ was administered via the diet. In such studies, E 171 was typically incorporated at very high concentrations into animal feed through simple admixing of the dry material. This is very different from the processes used in the food industry to accurately disperse E 171 as a suspension within human food matrices, which entail mechanical methods (such as high-shear mixing, colloid and disk mills and high-pressure homogenizers) delivering relatively high energies [14,49,50,51,52]. Only if E 171 is dispersed effectively do a sufficient number of particles lie in the size range 200–300 nm, where they can fulfil E 171’s purpose as a food additive.

A scientific principle incorporated in the EU regulatory safety assessment of small and nanoparticles is that the test conditions must consider worst-case scenarios, which means exposure to the most dispersed form, i.e., the form expected to result in the highest intestinal uptake. This principle is embedded in regulatory data requirements for proper and fresh dispersion of the material before testing, as well as confirmation thereof [8]. The EFSA guidance on the risk assessment of nanomaterials stipulates that when toxicological studies entail administration of the test item via the animal diet, the process for mixing the nanomaterial with the feed should mimic the conditions of consumer exposure to the nanoparticles, reflecting the expected level of agglomeration; this normally requires the test item to be suspended in a liquid medium beforehand by using an appropriate dispersion protocol [9]. The direct admixing of animal feed with E 171 as a powder (i.e., in the form in which particles are prone to the highest attractive forces and form very large agglomerates [53]) is clearly not representative of human exposure conditions. It is not surprising that the toxicological studies in which titanium dioxide was coarsely blended with animal diets were assigned the lowest score in the EFSA assessment of E 171 safety as a food additive in view of (i) the absence of a dispersion protocol, (ii) the use of extremely high doses only, and (iii) the lack of confirmation of exposure to the fraction of small particles [13].

## 5. Conclusions

In order to fill the knowledge gaps regarding the agglomeration behavior and fate of food-grade titanium dioxide in human GID, the development of a sound analytical approach is a necessary prerequisite. A number of analytical techniques exist for the physicochemical characterization of nanomaterials and materials containing a fraction of small particles, either as pristine materials or in suspension (including suspensions in complex media). Each technique has its own merits and limitations, and only a thorough multi-technique approach in which each analytical tool is used on a solid scientific ground, i.e., to address the proper measurand with a technically valid method while remaining mindful of the underlying physical principles, can ensure that the results are relevant and reliable from the perspective of their use for regulatory risk assessment.

By applying this reasoned approach, we demonstrated that E 171’s constituent particles are resistant to dissolution in the GIT, reaching the small intestine unchanged, and if taken up by cells, they are not degraded in simulated lysosomal conditions, which confirms their potential for bioaccumulation. Cell uptake in the intestinal epithelia is size-dependent. and the degree of agglomeration of E 171 particles in small intestinal conditions is key to either allowing the process to occur or to preventing it. Taking advantage of the unique element- and particle-specific capabilities of spICP-MS, we were able to show that, after simulated GID, at levels reflecting human exposure, E 171 is present in the small intestine with a dispersion degree similar to that obtained when dispersing the material in water by means of high-energy sonication. This means that humans are internally exposed to titanium dioxide particles within a size range whereby they can be absorbed by the small intestine and cross it to reach the systemic circulation. This evidence has important consequences in the assessment of E 171 safety following oral exposure. Toxicological studies where E 171 is administered via oral gavage or via drinking water after proper dispersion, via a valid dispersion protocol entailing high-energy sonication, are relevant for use in regulatory risk assessment. On the other hand, toxicological studies entailing administration of the test item via the animal diet are relevant only if the process for mixing the nanomaterial with the feed mimics the conditions for consumer exposure to the smaller particles, reflecting the expected level of agglomeration. This should always be supported using valid analytical data, and evidence of exposure at the different dose levels should be provided in order to demonstrate the relevance of the study for use in risk assessment. Fate studies in GIT conditions appears to be a viable approach to this end, and their use in combination with an assessment of internal (tissue) concentrations is advisable.

## Figures and Tables

**Table 1:** Instrumental parameters for spICP-MS analysis.

Instrumental Parameters	Operating Conditions
Power RF	1600 W
Nebulizer	Quartz concentric
Spray chamber	Cyclonic spray chamber
Flow nebulizer	0.99 L min^−1^
Peristaltic pump	−20 rps
Mode	Standard
QID	On
Selected masses	^47^Ti, ^48^Ti
Dwell time	100 µs
Sampling time	60 s
Transport efficiency range	7.51 ± 0.18%
Density	3.9 (g cm^−3^)
Mass fraction particle/analyte	1.67

**Table 2:** Summary of the descriptors of the number-based size distribution of E 171-a constituent particles as determined via TEM analysis (*n* = 916): median and mean values of the Fmin and Fmax; aspect ratio; number- and mass-based percentages of particles with a Fmin smaller than 100 nm and 250 nm.

Fmin		Fmax		Aspect Ratio		% of constituent Particles with Fmin		% of Mass Fraction with Fmin ^a^	
Median (nm)	Mean (nm)	Median (nm)	Mean (nm)	Median	Mean	<100 nm	<250 nm	<100 nm	<250 nm
79	95	114	137	1.4	1.5	64	98	9	71

^a^ Assuming that the particles are prolate ellipsoids (a-axis (Fmin) = b-axis (Fmin) < c-axis (Fmax)), the volume (V) of each particle was estimated as V = 4/3×a2×c. The mass (M) of each particle was calculated as: M = Vρ, where ρ = the anatase density (3.89 g/cm^3^) [12].

**Table 3:** Main descriptors of the number-based PSD of E 171-a suspended in water and in NaOH 0.1 mM as determined via spICP-MS. Size is expressed as ESD.

Sample	Mean (nm)	D10 (nm)	D50 (Median) (nm)	D99.5 (nm)	Particles <100 nm (%)	Particles <250 nm (%)
H_2_O						
0.1 mg/mL	191	68	188	505	19	77
0.7 mg/mL	189	77	185	490	17	78
NaOH 0.1 mM						
0.1 mg/mL	190	77	186	488	18	78
0.7 mg/mL	180	73	172	510	20	81

**Table 4:** Intensity-weighted mean hydrodynamic diameter (sd), average intensity and polydispersity index of E 171-a in aqueous suspension at different pHs determined via DLS.

-	Mean Hydrodynamic Diameter (nm)		Average Intensity (%)		Average PDI
-	Peak 1	Peak 2	Peak 1	Peak 2	
Water (pH 5.5)	337 ± 142	4872 ± 644	97.5	2.5	0.210
Citric acid 0.1 M, sodium citrate 0.1 M (pH 2.9)	373 ± 222	4780 ± 712	97.9	2.1	0.251
Phosphate buffer 10 mM (pH 7)	811 ± 316	5244 ± 434	98.5	1.5	0.198

**Table 5:** Main descriptors of the number-based PSD of E 171-a and real E 171-containing samples submitted to GID and sampled at two time points of the intestinal phase, as determined via spICP-MS. Size is expressed as ESD.

Sample	Mean Particle Diameter (nm)	D10 (nm)	D50 (Median) (nm)	D99.5 (nm)	Particles <100 nm (%)	Particles <250 nm (%)
E 171-a—fasted GID						
0.01 mg/mL, T = 0	196	90	186	513	14	75
0.01 mg/mL, T = 30	191	87	178	514	16	77
0.1 mg/mL, T = 0	198	72	187	535	21	71
0.1 mg/mL, T = 30	203	75	189	561	20	69
0.7 mg/mL, T = 0	260	80	256	639	15	48
0.7 mg/mL, T = 30	265	76	259	659	15	46
E 171-a—fed GID						
0.01 mg/mL, T = 0	181	79	168	509	20	81
0.01 mg/mL, T = 30	176	74	164	498	24	82
0.1 mg/mL, T = 0	194	78	187	513	18	75
0.1 mg/mL, T = 30	191	76	184	519	19	75
0.7 mg/mL, T = 0	226	92	223	566	12	62
0.7 mg/mL, T = 30	230	97	226	539	11	61
Chocolate candies						
0.15 mg/mL, T = 0	193	71	173	565	23	73
0.15 mg/mL, T = 30	193	72	172	578	23	73
Cappuccino						
0.54 mg/mL, T = 0	212	94	204	544	12	68
0.54 mg/mL, T = 30	210	95	199	516	12	68

## Data Availability

Data is contained within the article and Supplementary Material.

## Supplementary Materials

The following supporting information can be downloaded at: https://www.mdpi.com/article/10.3390/nano13131908/s1, Figure S1: Schematic overview of sample treatment before TEM imaging of pristine E 171 or multi-technique characterization of E 171 water suspensions; Figure S2: Schematic overview of sample treatment for fate studies and subsequent analytical determinations; Figure S3: Lysosomal digestion; Figure S4: TEM images of pristine 171-b; Figure S5: CLS size characterization of E 171-a; Figure S6: CLS size characterization of E 171-b; Figure S7: DLS results for E 171-a in MilliQ water; Figure S8: DLS results for E 171-a in citrate buffer; Figure S9: DLS results for E 171-a in phosphate buffer; Figure S10: DLS results for E 171-b in MilliQ water; Figure S11: DLS results for E 171-b in citrate buffer; Figure S12: DLS results for E 171-b in phosphate buffer; Figure S13: Zeta-potential measure for E 171-a in phosphate buffer; Figure S14: Zeta-potential measure for E 171-a in citrate buffer; Figure S15: Zeta-potential measure for E 171-a in phosphate buffer; Figure S16: Zeta-potential measure for E 171-b in citrate buffer; Figure S17: TEM images for E 171-a after fed gastrointestinal in vitro digestion; Figure S18: STEM images and corresponding EDX maps for E 171-a after fed gastrointestinal in vitro digestion; Table S1: Composition of Stock solutions of SSF, SGF, SIF, Bovine Bile and enzymes buffers; Table S2: Composition of lysosomal simulated fluid; Table S3: Mass recovery, expressed as titanium dioxide, for the 0; 24; 72 h time points of lysosomal digestion in glass and in falcon polypropylene tubes; Table S4: Instrumental parameters total titanium analysis; Table S5: Summary of the descriptors of the number based size distribution of E 171-b constituent particles as determined by TEM analysis; Table S6: Comparison between single isotope and merged 47Ti + 48Ti spICP-MS mass balances (%) for E 171-a; Table S7: Other descriptors of the number-based PSD of E 171-a suspended in water and in NaOH 0.1 mM as determined by spICP-MS (complementary parameters to Figure 3); Table S8: DLS pH dependent results for E 171-b; Table S9: Zeta potential results for E 171-a in citrate and phosphate buffer; Table S10: Zeta potential results for E 171-b in citrate and phosphate buffer; Table S11: Comparison of centrifugation vs filtration via syringe 5 μm filters as sample treatment for intestinal digestates before analytical measurements; Table S12: E 171-a recovery experiments: TiO2 recovery as determined via total Ti analysis by ICP-MS after MW digestion; Table S13: Other descriptors of the number-based PSD of E 171-a and real E 171-containing samples submitted to GID and sampled at two time points of the intestinal phase as determined by spICP-MS (complementary parameters to Figure 3). References [12,54] are cited in the supplementary materials.
