# Supplementary material for: Agglomeration Behavior and Fate of Food-Grade Titanium Dioxide in Human Gastrointestinal Digestion and in the Lysosomal Environment
Source: Nanomaterials (Basel). 2023 Jun 22;13(13):1908. doi: 10.3390/nano13131908 (PMC10343824; doi:10.3390/nano13131908)
Supplement: Supplementary file 1 [file nanomaterials-13-01908-s001.zip › nanomaterials-2451304-supplementary.pdf]

Supplementary information

Agglomeration behaviour and fate of food-grade titanium dioxide in human gastrointestinal digestion and in the lysosomal environment

Materials and Methods

Figure S1. Schematic overview of sample treatment before TEM imaging of pristine E 171 or multi-technique characterization of E 171 water suspensions

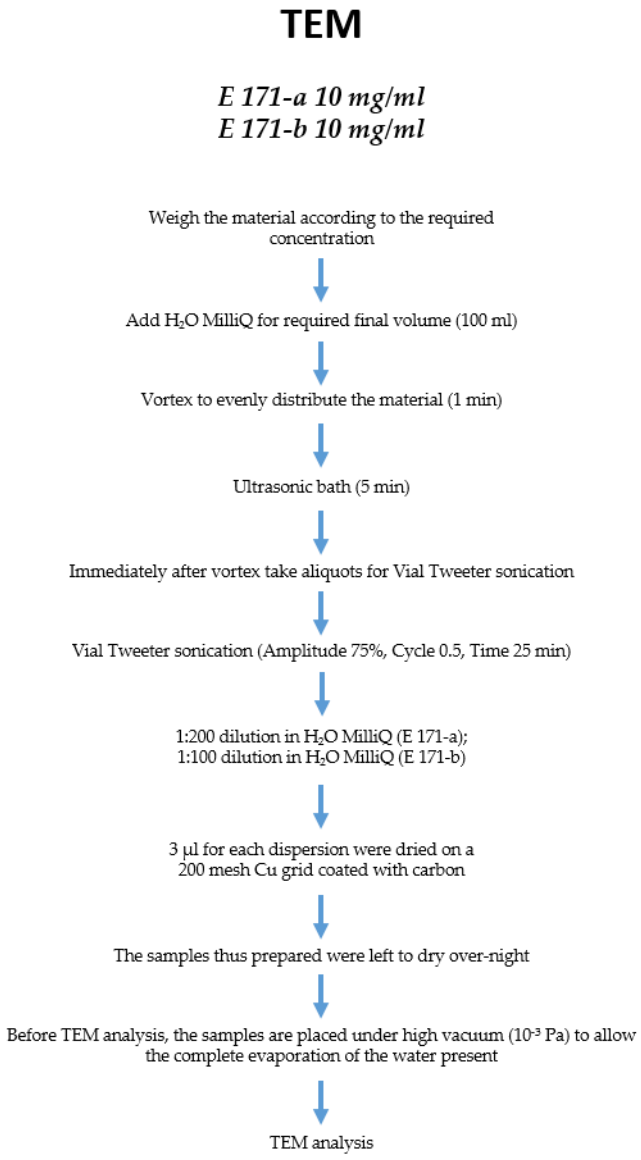

spICP-MS

E 171-a 0.1-0.7 mg/ml (MilliQ)

Weigh the material according to the required concentration

↓

Add H_2O MilliQ for required final volume (100 ml)

↓

Vortex to evenly distribute the material (1 min)

↓

Ultrasonic bath (5 min)

↓

Immediately after vortex take aliquots for Vial Tweeter sonication

↓

Vial Tweeter sonication (Amplitude 75%, Cycle 0.5, Time 25 min)

↓

Vortex to evenly distribute the material (1 min)

↓

Diluted with ultrapure water to achieve a particle concentration of 1000–2000 particles per 60 s scan time

↓

Agglomeration/ deagglomeration behavior (number based size distribution) by spICP-MS

spICP-MS

E 171-a 0.1-0.7 mg/ml (NaOH 0.1 mM)

Weigh the material according to the required concentration

Add NaOH 0.1 mM for required final volume (100 ml)

Vortex to evenly distribute the material (1 min)

Ultrasonic bath (5 min)

Immediately after vortex take aliquots for Vial Tweeter sonication

Vial Tweeter sonication (Amplitude 75%, Cycle 0.5, Time 25 min)

Vortex to evenly distribute the material (1 min)

Diluted with ultrapure water to achieve a particle concentration of 1000–2000 particles per 60 s scan time

Agglomeration/ deagglomeration behavior (number based size distribution) by spICP-MS

CLS

E 171-a 0.1 mg/ml
E 171-b 0.1 mg/ml

Weigh the material according to the required concentration

↓

Add H_2O MilliQ for required final volume (100 ml)

↓

Vortex to evenly distribute the material (1 min)

↓

Ultrasonic bath (5 min)

↓

Immediately after vortex take aliquots for Vial Tweeter sonication

↓

Vial Tweeter sonication (Amplitude 75%, Cycle 0.5, Time 25 min)

↓

1:100 dilution in H_2O MilliQ

↓

CLS analysis

DLS

E 171-a 0.1 mg/ml
E 171-b 0.1 mg/ml

Weigh the material according to the required concentration

↓

Add H_2O MilliQ or Phosphate buffer 10 mM or Citric acid/sodium citrate buffer, for required final volume (100 ml)

↓

Vortex to evenly distribute the material (1 min)

↓

Ultrasonic bath (5 min)

↓

Immediately after vortex take aliquots for Vial Tweeter sonication

↓

Vial Tweeter sonication (Amplitude 75%, Cycle 0.5, Time 25 min)

Water H_2O MilliQ; pH 5.5*	Phosphate buffer 10 mM; pH 7*	Citric acid/sodium citrate buffer; pH 2.89*
↓	↓	↓
DLS analysis	DLS analysis<br>Z-Potential analysis	DLS analysis<br>Z-Potential analysis

* pH is monitored by means of a pH-meter

Delivered energy

The delivered energy (J) applied during Vial Tweeter (indirect) sonication and ultrasonic bath of each sample was calculated as suggested by Mech et al [1] This SOP allows harmonization in the use of different sonication instruments and methods by determination of the actual energy effectively transferred to the test sample. Here the energy density (J g^{-1}) is reported, as this value is not dependent from the mass of the medium (MilliQ) used for calibration.

The experiment for vial tweeter calibration was performed in triplicate (amplitude 75%; cycle 0.5; 25 min; 1.5 g MilliQ) and the resulting energy density was 115 J g^{-1}.

The experiment for ultrasonic bath calibration was performed in triplicate (10 min each; 100 g MilliQ) and the resulting energy density was 11 J g^{-1}.

Figure S2. Schematic overview of sample treatment for fate studies and subsequent analytical determinations

E 171-a fed GI digestion (10 mg/ml) for TEM analysis

Weigh the material according to the required concentration

↓

Add H_2O MilliQ for required final volume (100 ml)

↓

Vortex to evenly distribute the material (1 min)

↓

Ultrasonic bath (5 min)

↓

Immediately after vortex take aliquots for Vial Tweeter sonication

↓

Vial Tweeter sonication (Amplitude 75%, Cycle 0.5, Time 25 min)

↓

1:200 dilution in H_2O MilliQ (E 171-a);

↓

An aliquot of the dispersion is added to the model food (2 h of contact at room temperature); therefore, simulated salivary fluid (2 min, 37 °C pH ~ 7), followed by simulated gastric fluid (2 h, 37 °C, pH ~ 2-3) is added

↓

Intestinal fluid is added and after 30 min from the beginning of the intestinal step (37 °C, pH ~ 6,5-7), an aliquot of the digested product is taken and filtered through glass filters, pore size 2 μm

↓

The filtrate, placed in a 50 mL falcon, is centrifuged for 10 min at 4200 rpm (3392 g)

↓

The formation of three phases is observed: supernatant, intermediate part and pellet.

↓

The pellet must be purified from the matrix (enzymes, salts, micelles, fats) and concentrated before analysis TEM. The pellet purification operation is accomplished through washing with organic solvents and water

↓

Washing with DMSO

↓

Washing with EtOH

↓

Washing with cyclohexane

↓

Another washing with DMSO

↓

Washing with H_2O MilliQ

↓

3 μl for each dispersion is dried on a 200 mesh Cu grid coated with carbon

↓

The samples thus prepared is left to dry over-night

↓

Before TEM analysis, the samples are placed under high vacuum (10^{-3} Pa) to allow the complete evaporation of the water present

↓

TEM analysis

* During digestion the samples are under constant stirring into a temperature-controlled incubation chamber GFL 1083. pH is monitored by means of a pH-meter

*E 171-a fed/fasted GI digestion**
(0.01-0.1-0.7 mg/ml)

Weigh the material according to the target concentration (0.01-0.1-0.7 mg/ml)

↓

Add MilliQ H_2O until final volume (100 ml)

↓

Vortex (1 min)

↓

Ultrasonic bath to evenly distribute the material (5 min)

↓

Immediately after vortex take aliquots for Vial Tweeter Sonication

↓

Vial Tweeter sonication (Amplitude 75%, Cycle 0.5, Time 25 min)

↓

Fasted digestion: Take 3 ml of E 171 dispersion for each replicate (x3)
Fed digestion: add 3 ml of E171 dispersion to 3 g of food and wait 2 h of contact between food-E 171, room temperature (x3)

↓

Add reagents for salivary step (2 min), 37 °C, pH ~ 7

↓

Add reagents for gastric step (2 h), 37 °C, pH ~ 2-3

↓

Add reagents for intestinal step and sample at time points 0; 5; 10; 15; 30; 60 min, 37 °C, pH ~ 6.5-7

↓

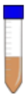

Vortex the time point aliquot; 1:100 dilution; 5 µm filtration

- All the time points: dissolution test (ultrafiltration) → ICP-MS total titanium analysis of the ultrafiltered fraction
- T=0; 30 min: agglomeration/ deagglomeration behavior (number based size distribution) by spICP-MS
- T=0; 30 min: microwave-supported oxidative digestion → ICP-MS total titanium analysis

* During digestion the samples are under constant stirring into a temperature-controlled incubation chamber GFL 1083. pH is monitored by means of a pH-meter

E 171-b fasted GI digestion 0.01 mg/ml*
E 171-c fasted GI digestion 0.01-0.1-0.7 mg/ml*

Weigh the material according to the required concentration

↓

Add H_2O MilliQ for required final volume (100 ml)

↓

Vortex to evenly distribute the material (1 min)

↓

Ultrasonic bath (5 min)

↓

Immediately after vortex take aliquots for Vial Tweeter sonication

↓

Vial Tweeter sonication (Amplitude 75%, Cycle 0.5, Time 25 min)

↓

Take 3 ml of E 171 dispersion for each FASTED replicate (x3)

↓

Add reagents for salivary step (2 min), 37 °C, pH ~ 7

↓

Add reagents for gastric step (2 h), 37 °C, pH ~ 2-3

↓

Add reagents for intestinal step and sample at time points 0; 5; 10; 15; 30; 60 min, 37 °C, pH ~ 6,5-7

↓

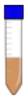

Vortex of the time point aliquot; 1:100 dilution; 5µm filtration

↙ All the time points: dissolution test (ultrafiltration)

↓

ICP-MS total titanium analysis of the ultrafiltered fraction

↘ T=0; 30 min: microwave-supported oxidative digestion

↓

ICP-MS total titanium analysis

* During digestion the samples are under constant stirring into a temperature-controlled incubation chamber GFL 1083. pH is monitored by means of a pH-meter

*Chocolate candies**

Weigh 3 yellow dragees

↓

Add 25 ml of SSF, stirred by hand until the outer coating was solubilised and only the chocolate from the dragees was visible

↓

Remove the chocolate core

↓

Add another 10 ml of SSF

↓

Ultrasonic bath (5 min)

↓

Take 5.4 ml of the dispersion and add the enzymes for the 2 min salivary step, 37 °C, pH ~ 7

↓

Add reagents for gastric step (2 h), 37 °C, pH ~ 2-3

↓

Add reagents for intestinal step and sample at time points 0; 5; 10; 15; 30; 60 min, 37 °C, pH ~ 6,5-7

↓

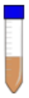

Vortex of the time point aliquot; 1:100 dilution; 5µm filtration

- All the time points: dissolution test (ultrafiltration) → ICP-MS total titanium analysis of the ultrafiltered fraction
- T=0; 30 min: spICP-MS
- T=0; 30 min: microwave-supported oxidative digestion → ICP-MS total titanium analysis

* During digestion the samples are under constant stirring into a temperature-controlled incubation chamber GFL 1083. pH is monitored by means of a pH-meter

*Powdered cappuccino**

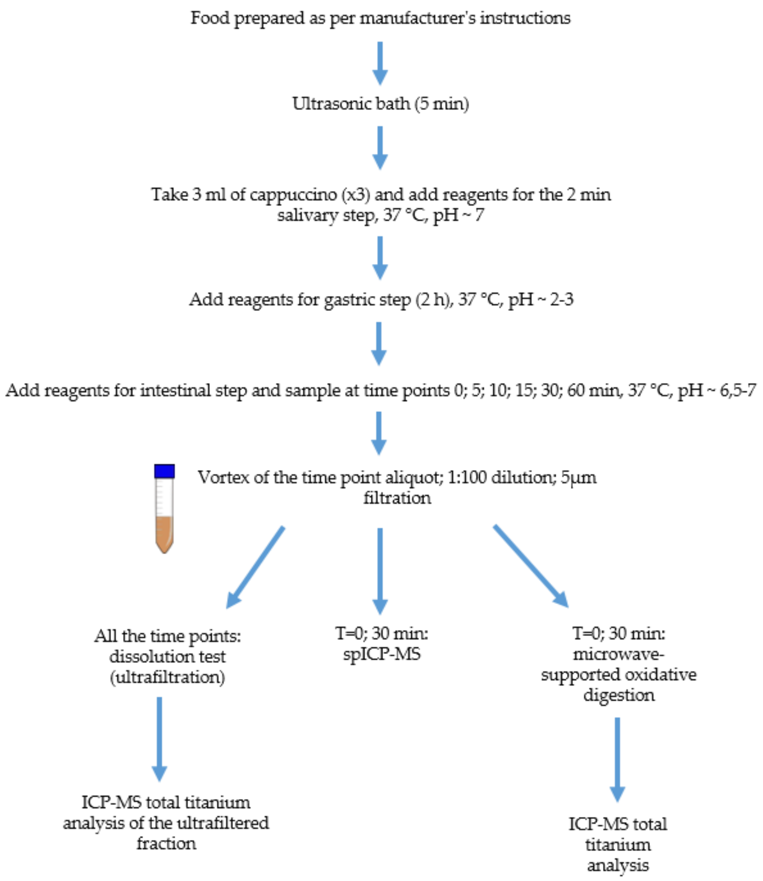

* During digestion the samples are under constant stirring into a temperature-controlled incubation chamber GFL 1083. pH is monitored by means of a pH-meter

E 171-a lysosomal digestion 0.1 mg/ml

Weigh the material according to the required concentration

↓

Add H_2O MilliQ for required final volume

↓

Vortex to evenly distribute the material (1 min)

↓

Ultrasonic bath (5 min)

↓

Immediately after vortex take aliquots for Vial Tweeter sonication

↓

Vial Tweeter sonication (Amplitude 75%, Cycle 0.5, Time 25 min)

↓

Take 3 ml of E 171 dispersion for each lysosomal replicate (x3)

↓

Add 27 ml of lysosomal simulated fluid for each sample, 37 °C, pH ~ 4.5-5*

↓

Take an aliquot of the sample at time points 0; 2; 5; 24; 72 h

↓

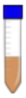

Vortex of the time point aliquot; 1:20 dilution; 5µm filtration

↙ ↘

All the time points: dissolution test (ultrafiltration)

↓

ICP-MS total titanium analysis of the ultrafiltered fraction

T=0; 24; 72 hours: microwave-supported oxidative digestion

↓

ICP-MS total titanium analysis

* During digestion the samples are under constant stirring into a temperature-controlled incubation chamber GFL 1083. pH is monitored by means of a pH-meter

Determination of the size LOD in spICP-MS measurements

In spICP-MS measurements the smallest pulse height that can be distinguished from the background determines the smallest detectable particle mass that can be related to the smallest detectable particle size. The instrumental size LOD was calculated on daily basis as the smallest particle distinguishable from the background and it ranged from 27 nm to 39 nm.

Table S1. Composition of Stock solutions; SSF; SGF; SIF; Bovine Bile and enzymes buffers

**Stock solution (Vf = 200mL)**		
	conc (M)	weighted (g)
KCl	0.5	7.46
KH_2PO_4	0.5	13.6
$NaHCO_3$	1	16.8
NaCl	2	23.4
$MgCl_2 * 6H_2O$	0.15	6.1
$(NH_4)_2CO_3$	0.5	9.6
NaOH	1	8
HCl	6	-
$CaCl_2 * 2H_2O$	0.03	0,88
**Simulated salivary fluid SSF (Vf=200mL)**		
	conc. in SSF (mM)	mL stock sol.
KCl	15.1	6.04
KH_2PO_4	3.7	1.48
$NaHCO_3$	13.6	2.72
NaCl	-	-
$MgCl_2 * 6H_2O$	0.15	0.2
$(NH_4)_2CO_3$	0.06	0.024
NaOH	-	-
HCl	1.1	0.036
**Simulated gastric fluid SGF (Vf=200mL)**		
	conc. in SGF (mM)	mL stock sol.
KCl	6.9	2.76
KH_2PO_4	0.9	0.36
$NaHCO_3$	25	5
NaCl	47.2	4.72
$MgCl_2 * 6H_2O$	0.1	0.16
$(NH_4)_2CO_3$	0.5	0.2
NaOH	-	-
HCl	15.6	0.52
**Simulated intestinal fluid SIF (Vf=200mL)**		
	conc. in SIF (mM)	mL stock sol.
KCl	6.8	2.72
KH_2PO_4	0.8	0.32
$NaHCO_3$	85	17

NaCl	38.4	3.84
$MgCl_2 * 6H_2O$	0.33	0.44
$(NH_4)_2CO_3$	-	-
NaOH	-	-
HCl	8.4	0.28

Enzymes

	Effective Activity (U/mg)	Desired activity (U/mL)	mg	Vf (mL)
α-amilase	478	1500	6,28	2
pepsin*	3772	25000	36.7	2
Gastric lipase	16		21	1.4
pancreatin	100	800	104	13

* Only 93% is protein. [Stable solution for 2-3 months if stored at pH = 4.4 and T = -20 ° C]

Bovine Bile Vf=7mL (SIF)

	conc. (M)	weighted (g)
Bovine Bile B3883	0,16	1.440

Bovine Bile B3883 lot # SLCD5439

Gastric Lipase + Pepsin

Gastric Lipase	6	U/ml final		
Supplier	Lipolytech			
lot number	RGE15			
U/mg solid	72	U needed at	240	U/ml
weight needed	4.5	mg		
weighed	15.00	mg of RGE for 1 tube		
concentration	15.00	mg/ml in water		
volume of water	1000	ml		
Pepsin	2000	U/ml final		
Pepsin Activity U/mg RGE solid	720			
Total Pepsin activity needed	24000	U needed at	80000	U/ml
Pepsin Activity U/mg provided by RGE	3240	U		
Sigma pepsin P7012				
lot number	SLBT8881			
Activity U/mg solid	3772			
Pepsin activity U/mg provided by pepsin	20760	U needed at	69200	U/ml
weight needed	5,50	mg		
weighed	18.35	mg of Pepsin		
concentration	18.35	mg/ml in water		
volume of water	1000	ml		

α-amilase: Lot. N. BCBD1452V; Cod 1069 1g
Pepsin: Lot. N. SLBT8881; Cod P7012 5g
Pancreantin: Lot. N. SLBF6528V; Cod P1750 100g
Gastric lipase: (https://www.lipolytech.com/catalogue1) RGE 15 lot. N. 1403

Table S2. Composition of lysosomal simulated fluid

**Simulated lysosomal fluid (composition for 1L of buffer)**		
	g	conc (mM)
Citric acid	20.8	108.3
NaCl	3.21	54.9
NaOH	6	150
$CaCl_2$	0.097	0.874
$Na_3PO_4 * 7H_2O$	0.179	0.617
Na_2SO_4	0.039	0.276
$MgCl_2 * 6H_2O$	0.106	0.522
sodium citrate * $2H_2O$	0.077	0.262
Sodium lactate	0.085	0.786
Glycine	0.059	0.786

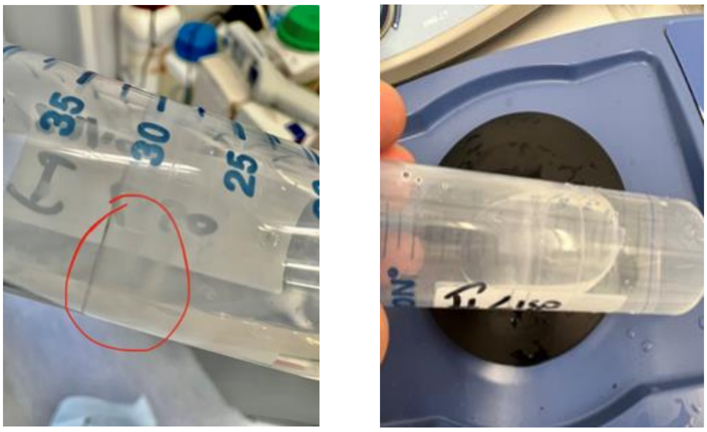

Figure S3. Lysosomal digestion. White corona on the plastic wall of the polypropylene Falcon tube at the liquid-air interface. The phenomenon was observed only for the samples containing E 171 and not in the procedural blanks, suggesting that the corona was made from adsorbed E 171 particles. As a consequence, the E 171 mass recovery after lysosomal in vitro digestion in polypropylene tubes was markedly decreased. Although in glass the white corona was not visible, massive adsorption did occur leading to very low and variable recoveries. Thus, it is recommended to use polypropylene tubes in lysosomal digestion, being mindful of checking the formation of the corona over time and, if present, to recover the TiO_2 adsorbed (e.g. scraping the walls of the tube with a titanium-free spatula).

Table S3. Mass recovery, expressed as titanium dioxide, for the 0; 24; 72 h time points of lysosomal digestion in glass and in falcon polypropylene tubes. The recovery is referred to the initial amount of titanium dioxide obtained by ICP-MS analysis of the microwave digested E171 dispersion. The 72 h time point in polypropylene tube was analyzed after having scraped the walls of the tube with a titanium-free spatula (mass recovery improved with this treatment).

**E 171-a 0.1 mg/ml**	**Rec. (%)**	
	Glass	Polypropylene tube
T=0	28	52
T=24 hours	21	33
T=72 hours	32	79

ICP-MS analytical measurements

Microwave digestion

2 g of T=0 and 30 minutes subsamples were placed in high-pressure Teflon containers with 1.5 ml of HNO_3 67-69% v/v (ultrapure grade, Carlo Erba, Rodano, Italy), 0.2 ml HF (ultrapure grade, Carlo Erba, Rodano, Italy), and 0.5 ml of H_2O_2 30% v/v (ultrapure grade, Sigma-Aldrich, Darmstadt, Germany). Samples were digested in a microwave system (UltraWave Single Reaction Chamber Microwave Digestion System, Milestone, Bergamo, Italy) with the following program: 23 min 240 °C ramp, 10 min 240 ° C hold (maximum power 1400 W), 30 min depressurization and cooling at room temperature. Then the samples were diluted up to 7.5 g with MilliQ water and stored in fridge until total titanium analysis.

Ultrafiltration

Three g of every fraction collected were placed in Amicon 50 KDa ultrafiltration tubes. The samples were centrifuged under the following conditions: 6 minutes, 6000 rpm, RT for fed GID; 3 minutes, 6000 rpm, RT, for fasted GID. Samples were then acidified to reach 1% HNO_3 and then stored in fridge until total titanium analysis.
Amicon 50KDa filters with a nominal size cut-off of 5 nm (https://www.sigmaaldrich.com/IT/en/technical-documents/technical-article/analytical-chemistry/filtration/nanoparticle-ultrafiltration), have been used for separation of particulate and dissolved fractions.

Total titanium analysis

Total titanium analyses were conducted by means of an ICP-QQQ mass spectrometer (Agilent 8800, Agilent Technologies Inc., Tokyo, Japan). The instrument is equipped with an octopole-based collision/reaction cell (ORS3 cell) in between the two-quadrupole analyzers and operated in MS/MS mode and single quadrupole mode, the analytical masses monitored in MS/MS mode ^{47}Ti, ^{48}Ti, ^{49}Ti, ^{50}Ti and in single quad. ^{47}Ti, ^{48}Ti, ^{49}Ti, ^{50}Ti. The sample introduction system of the ICP mass spectrometer was replaced with inert (non-quartz) components, a 2.5 mm ID sapphire injector and a PFA concentric nebulizer with a double-pass PFA spray chamber cooled down to 2 °C. Based on the fine-tuning of the method on the samples used to develop it, the best mass was ^{48}Ti on MS/MS mode. The described results are obtained using this mass. Instrument operational conditions are given in Table S4.

When necessary, the system was operated also in MS/MS mode by using NH_3/He (10/90) as a reaction gas which provided interference-free conditions. Analytical masses were set at 48 →150 for titanium and 103→103 for rhodium (used as internal standard) on the first quadrupole Q1 and the second quadrupole Q2, respectively.

The system was tuned daily in order to reach maximum sensitivity of Ti and quantitative determinations were carried out by external calibration. The standard solution for the different elements and internal standard solution were obtained by diluting the stock solution (1 g L^{-1}) (High-Purity, Charleston, SC, USA) with high-purity deionized water obtained from a Milli-Q Element system (Millipore, Molsheim, France). An external calibration curve for Ti (0.25-10 µg L^{-1}) were prepared with the corresponding amount of HNO_3 based on the

dilution of the samples and a constant addition of 0.5 μg L^{-1} of Rh, the internal standard selected for minimizing errors associated with the response provided by the device. The LODs (LOQs) achieved, based on the 3σ (6 σ) criterion where σ is the standard deviation of the measurement of 30 method blanks, were in the order of were in the order of 0.13 μg/kg (0.26 μg/kg). Each reagent used for the sample mineralization (HNO_3; H_2O_2; HF) was analyzed individually to detect each specific contribution to the method's blank.

Table S4: Instrumental parameters total titanium analysis

Instrumental parameters	Operating conditions
Power RF	1550 W
Nebulizer	Esi PFA-LC
Spray Chamber	Scott PFA inert kit
Flow nebulizer	0.82 L min^{-1}
Makeup gas	0.31 L min^{-1}
Peristaltic pump	0.15 rps
Mode	MS/MS, single quad, NH_3 (15%)
Sampling period	30 sec
Integration time	2 sec
Selected masses Q1	^{47}Ti, ^{48}Ti, ^{49}Ti, ^{50}Ti, ^{103}Rh
Selected masses Q2	^{47}Ti, ^{48}Ti, ^{48}Ti->^{150}Ti, ^{49}Ti, ^{50}Ti, ^{103}Rh

Results

Pristine materials characterization

TEM analysis

E 171-b consists of anatase particles of mixed morphology including spheres and particles with irregular geometry consisting of polyhedrons (i.e. with a strong angular component). The constituent particles have a prevalent Fmin diameter of 40-100 nm and are surrounded by an organic coating with a thickness of 2-3 nm. The descriptors of the number based distribution particle size distribution of E 171-b are summarized in Table S5.

Table S5. Summary of the descriptors of the number based size distribution of E 171-b constituent particles as determined by TEM analysis (n = 321): median and mean values of the Fmin, Fmax; Aspect ratio; Number - and mass-based percentages of particles with a minimum Feret diameter smaller than 100nm and 250 nm. Assuming that the particles are prolate ellipsoids [a-axis (Fmin) = b-axis (Fmin) < c-axis (Fmax)], the volume (V) of each particle was estimated $V = 4/3*\pi*a^2*c$. The mass (M) of each particle was calculated as: $M=V\varrho$ with ϱ = the density of the particles (3.89 g/cm^3), the calculated bulk density of anatase TiO_2 [2]

Fmin		Fmax		Aspect ratio		% of constituent particles with Fmin		% of mass fraction with Fmin	
Median (nm)	Mean (nm)	Median (nm)	Mean (nm)	Median	Mean	< 100 nm	< 250 nm	<100 nm	< 250 nm
94	108	136	145	1.5	1.4	53	98	11	80

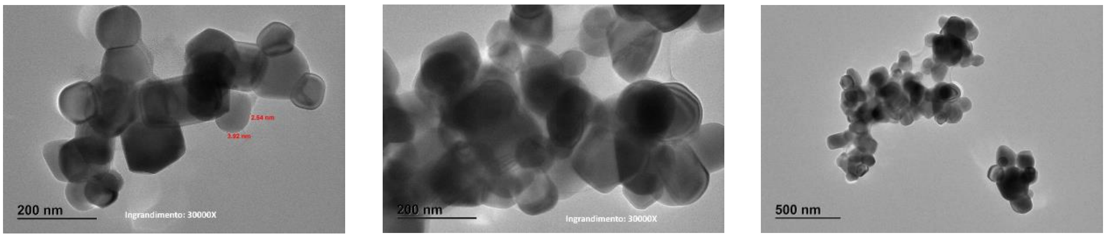

(a) (b) (c)

Figure S4. TEM images of pristine 171-b (**a**) 30000X magnification; (**b**) 30000X magnification; (**c**) 10000X magnification.

Single particle ICP-MS analysis

Integration of ^{47}Ti and ^{48}Ti data to obtain a single E171 PSD

In order to create a single E171 PSD, ^{47}Ti and ^{48}Ti data were merged. The criterion for combining the PSDs obtained by monitoring the two isotopes was as follows.

Each distribution obtained by the two isotopes in isolation was represented by selecting a bin equal to 10. The left-hand side of the combined distribution was built using ^{48}Ti. Then the ^{47}Ti frequency class showing frequencies higher than ^{48}Ti and where such frequencies remained higher for 5 subsequent frequency classes was identified. The final merged distribution was the built using ^{48}Ti data below this size threshold and ^{47}Ti data above this size threshold. This means that ^{47}Ti frequencies were used to obtain the right-hand side of the combined distribution.

Table S6. Comparison between single isotope and merged ^{47}Ti + ^{48}Ti spICP-MS mass balances (%) for E 171-a. Ratios of spICP-MS particles mass balances to actually measured total Ti concentrations for the same sample (for simulated GID: 30 min time point, 0.1 mg/ml concentration), the latter measured by ICP-MS after MW digestion.

Sample	Mass balance ^{48}Ti (%)	Mass balance ^{47}Ti (%)	Mass balance ^{47}Ti + ^{48}Ti (%)
E 171-a pristine	38	62	70
E 171-a fasted	40	62	105
E 171-a fed	42	64	79
Chocolate candies	45	62	69
Powdered cappuccino	52	74	83

Table S7. Other descriptors of the number-based PSD of E 171-a suspended in water and in NaOH 0.1 mM as determined by spICP-MS (complementary parameters to Figure 3). Size is expressed as ESD.

Sample	Smallest Particle Diameter, Lower bound (D0) [nm]	Most Frequent Particle Diameter (Mode) [nm]*	D99.8 [nm]	Largest Particle Diameter, Upper bound (D100) [nm]
0.1 mg/ml (H_2O)	32	210	570	696
0.7 mg/ml (H_2O)	32	154	537	613
0.1 mg/ml (NaOH 0.1 mM)	32	218	537	610
0.7 mg/ml (NaOH 0.1 mM)	32	134	575	653

* In the presence of multimodal distributions, the value with the highest occurrence frequency was used for the evaluation of the results

CLS analysis

The mass-based arithmetic mean diameter determined from E 171-b PSDs at 0.1 mg TiO_2/ml was 229 nm. Graphical representations of the PSDs obtained by means of CLS for both E 171-a and E 171-b are shown below (Figures S5-S6).

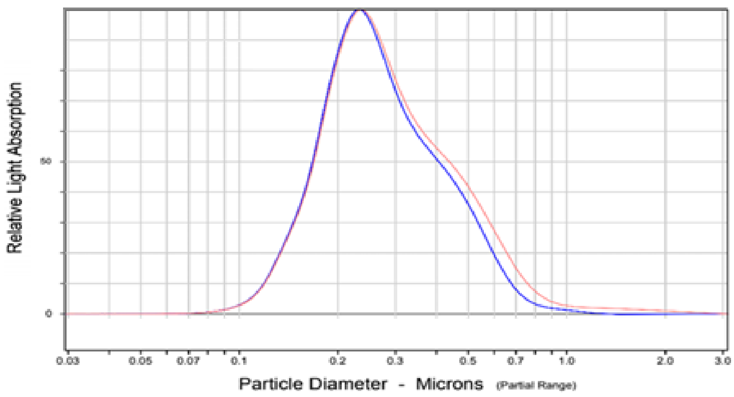

Figure S5. CLS size characterization of E 171-a. Data are mass-based arithmetic mean diameters.

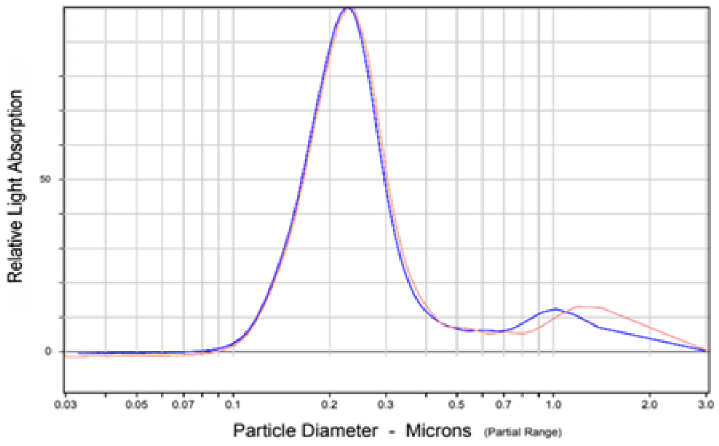

Figure S6. CLS size characterization of E 171-b. Data are mass-based arithmetic mean diameters.

DLS analysis

Table S8. DLS pH dependent results for E 171-b

	Average mean size (nm)		Average intensity (%)		Average PDI
	**Peak 1**	**Peak 2**	**Peak 1**	**Peak 2**	
E 171-b (pH 5.5)	308 ± 96	5191 ± 478	98.6	1.4	0.201
E 171-b (pH 2.9)	266 ± 78	5227 ± 458	99.3	0.7	0.199
E 171-b (pH 7)	606 ± 177	5505 ± 104	97.9	2.1	0.337

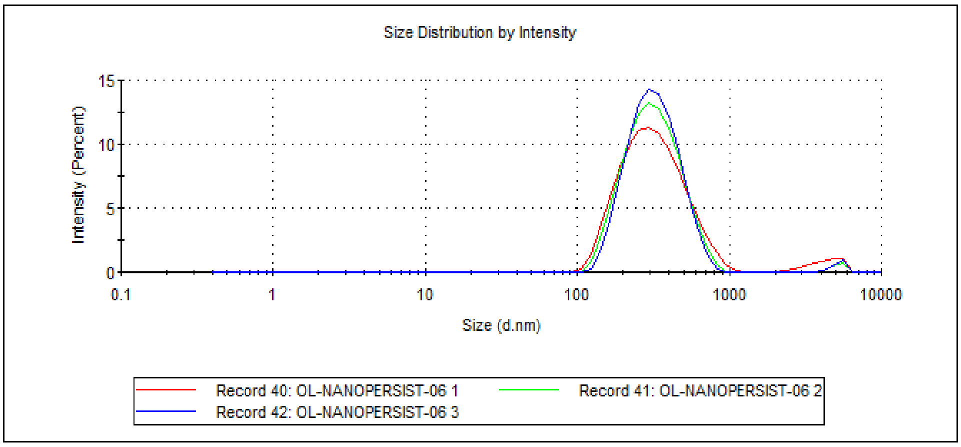

Figure S7. DLS results for E 171-a in MilliQ water.

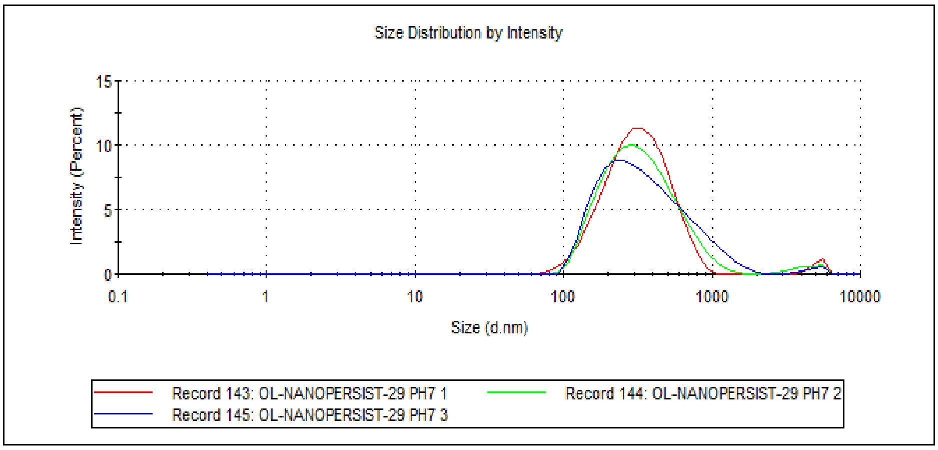

Figure S8. DLS results for E 171-a in citrate buffer.

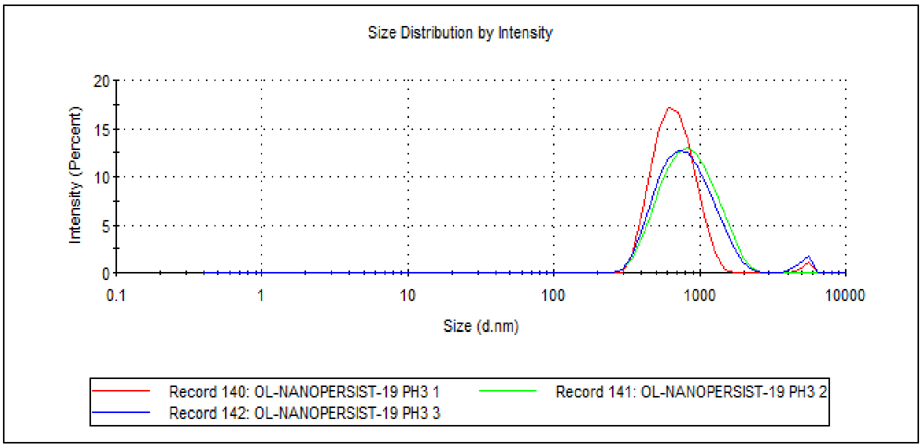

Figure S9. DLS results for E 171-a in phosphate buffer.

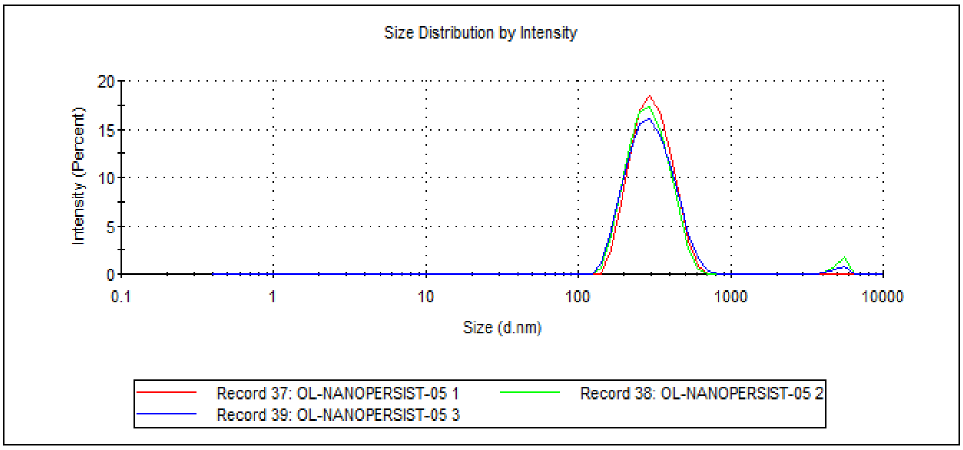

Figure S10. DLS results for E 171-b in MilliQ water

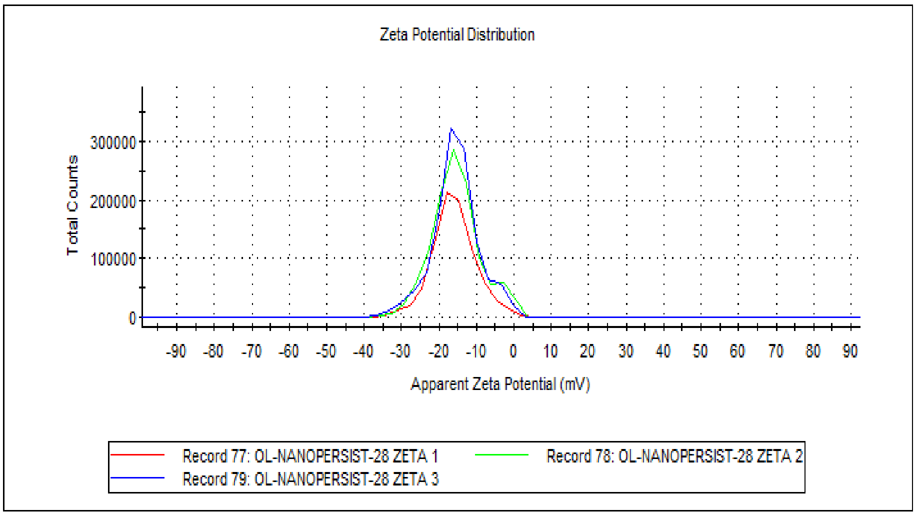

Figure S11. DLS results for E 171-b in citrate buffer

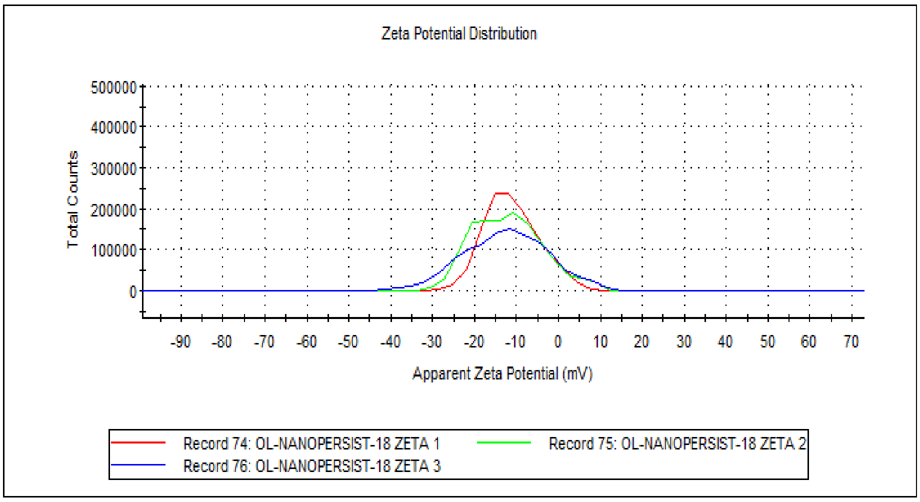

Figure S12. DLS results for E 171-b in phosphate buffer.

Zeta potential

For E 171-a, IEP measurements showed that the zeta-potential in citrate buffer was ca. -38 mV that changed to -57 mV in phosphate buffer (Table S9; Figure S13; Figure S14). For E 171-b the values were -12 mV and -16 mV, respectively (Table S10; Figure S15; Figure S16). In the latter case, the potential is, in absolute terms, smaller, which appears to be due to the organic coating of the material.

Table S9. Zeta potential results (3 runs) for E 171-a in citrate and phosphate buffer

	Z-potential (mV)	St. Dev. (mV)	Conductivity (mS/cm)
**Phosphate buffer**			
run 1	-54.0	5.00	1.12
run 2	-58.9	5.31	1.19
run 3	-60.2	4.80	1.24
**Citrate buffer**			
run 1	-35.7	3.93	2.92
run 2	-38.5	4.28	3.38
run 3	-39.7	7.24	3.56

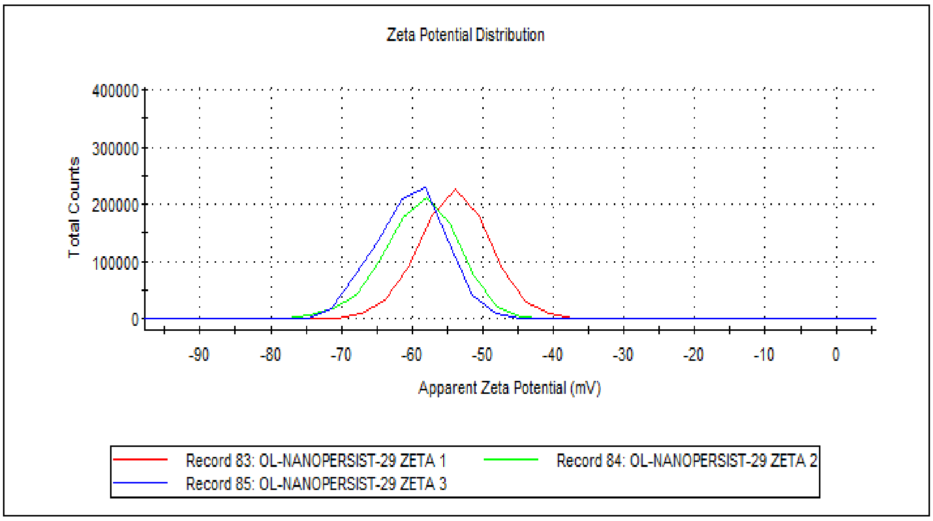

Figure S13. Zeta-potential measure for E 171-a in phosphate buffer

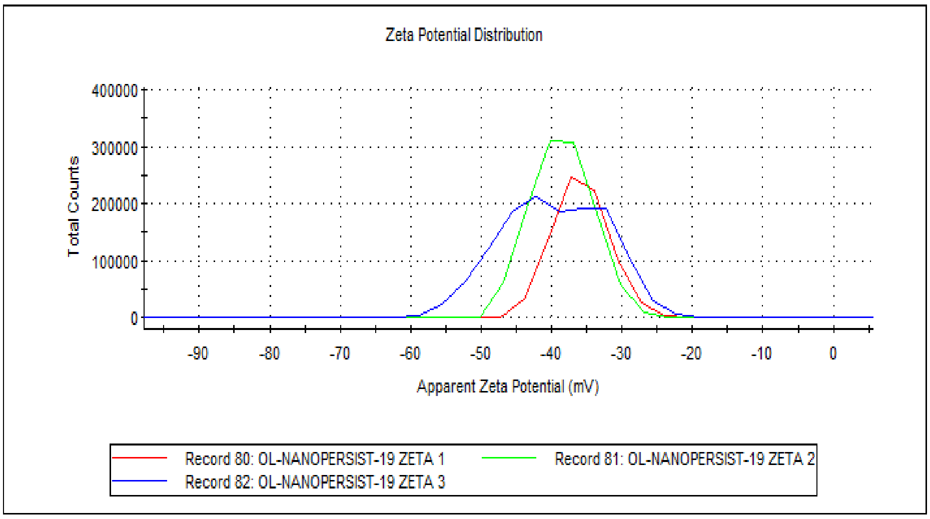

Figure S14. Zeta-potential measure for E 171-a in citrate buffer

Table S10. Zeta potential results (3 runs) for E 171-b in citrate and phosphate buffer

	Z-potential (mV)	St. Dev. (mV)	Conductivity (mS/cm)
**Phosphate buffer**			
run 1	-16.0	5.83	1.11
run 2	-15.4	6.54	1.18
run 3	-15.5	6.24	1.22
**Citrate buffer**			
run 1	-11.1	6.37	2.91
run 2	-	-	-
run 3	-12.5	9.8	3.54

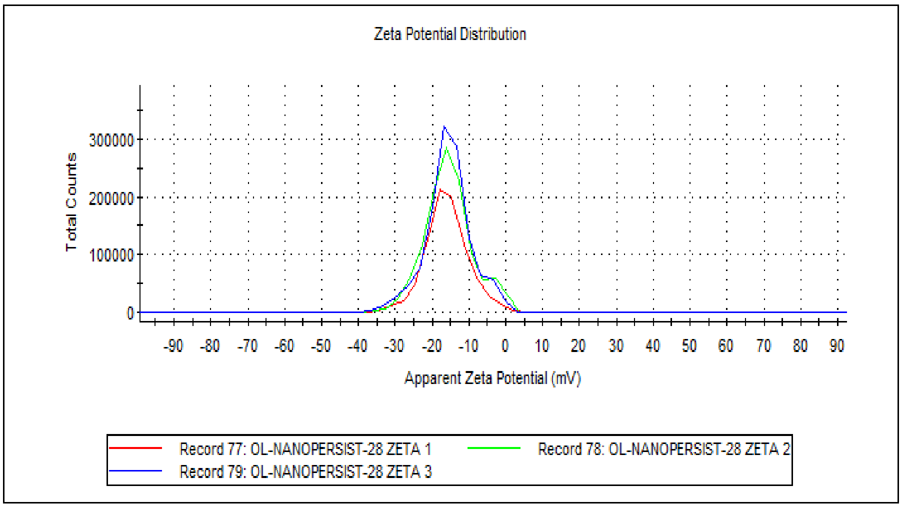

Figure S15. Zeta-potential measure for E 171-a in phosphate buffer

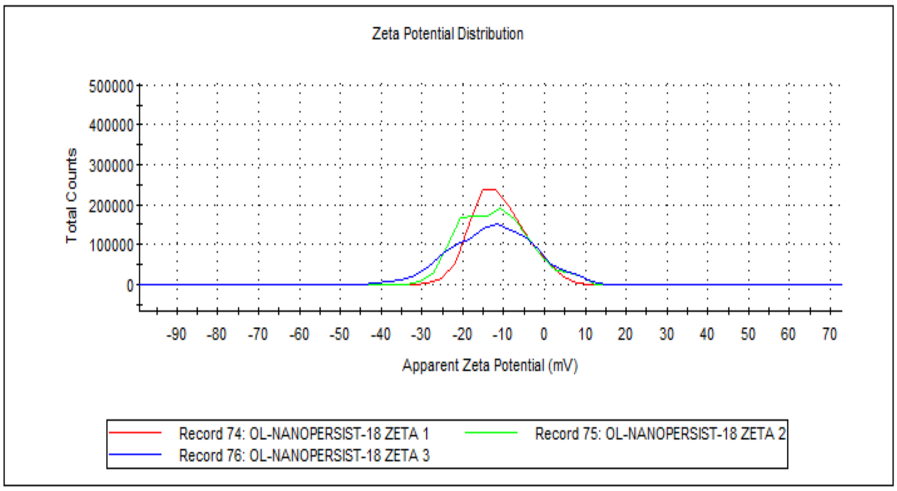

Figure S16. Zeta-potential measure for E 171-b in citrate buffer

Simulated gastrointestinal digestion

Table S11. Comparison of centrifugation *vs* filtration via syringe 5 μm filters as sample treatment for intestinal digestates before analytical measurements. The concentration values are reported as the average of three different experimental replicates and are expressed as titanium dioxide.

	Ultrawave digested samples after centrifugation (mg/kg)	Ultrawave digested samples after filtration (mg/kg)	Theoretical concentration (mg/kg)
E 171-a fed GID	16.3	80.2	100

Table S12. E 171-a recovery experiments: TiO_2 recovery as determined via total Ti analysis by ICP-MS after MW digestion. Results are the average of 3 experimental replicates. For the pristine material, the actual concentration in dispersion is measured. For the 5 μm filtration, the filtration effect in MQW is checked. For the GID (fed and fasted conditions), the 5 μm filtration effect is checked in the presence of GIT simulated fluids (and, for the fed protocol, of the model food). In brackets, the % of recovery referred to the E 171-a initial concentration is shown.

	**Average concentration after MW digestion (mg/L TiO_2)**		
**Sample**	**Theoretical concentration: 10 mg/L**	**Theoretical concentration: 100 mg/L**	**Theoretical concentration: 700 mg/L**
E 171-a pristine	12	107	683
E 171-a (5 μm filtration)	5	18	514
**Fed GID**			
T=0	10 ± 3 (85%)	85 ± 3 (85%)	504 ± 9 (72%)
T=30 min	9 ± 2 (72%)	79 ± 7 (73%)	506 ± 35 (72%)
**Fasted GID**			
T=0	7 ± 1 (59%)	66 ± 6 (63%)	232 ± 26 (35%)
T=30 min	7 ± 1 (72%)	45 ± 1 (43%)	163 ± 52 (25%)

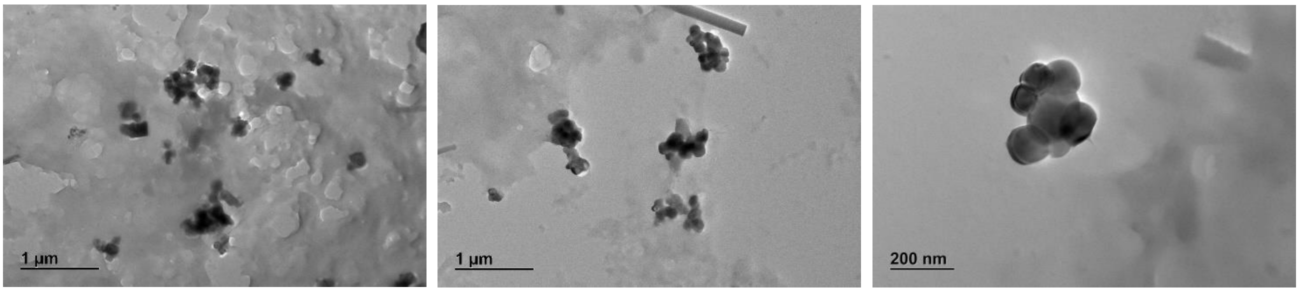

(a) (b) (c)

Figure S17. TEM images for E 171-a after fed gastrointestinal in vitro digestion (**a**) 5000X magnification; (**b**) 5000X magnification; (**c**) 20000X magnification.

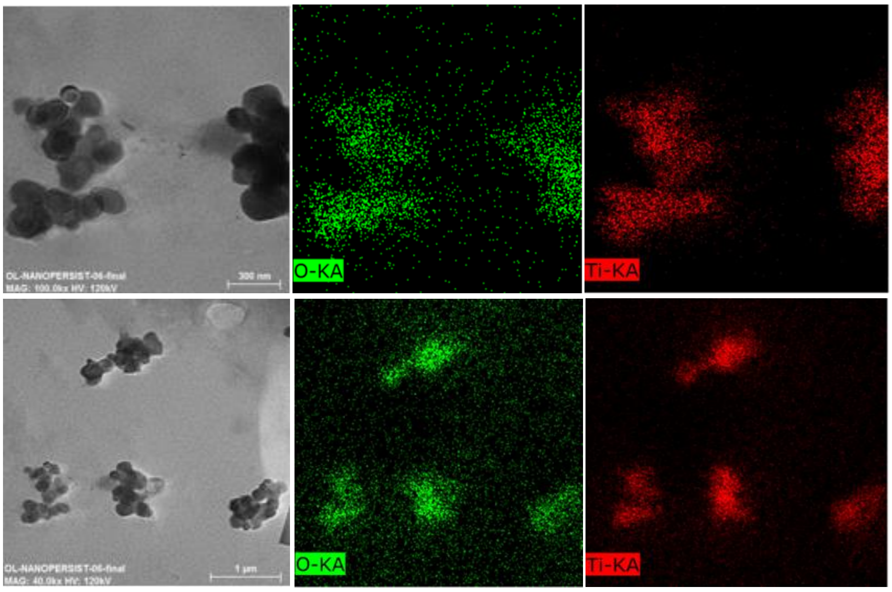

Figure S18. STEM images and corresponding EDX maps for E 171-a after fed gastrointestinal in vitro digestion.

Agglomeration behaviour in the intestinal phase

Table S13. Other descriptors of the number-based PSD of E 171-a and real E 171-containing samples submitted to GID and sampled at two time points of the intestinal phase as determined by spICP-MS (complementary parameters to Figure 3). Size is expressed as ESD.

Sample	Smallest Particle Diameter, Lower bound (D0)[nm]	Most Frequent Particle Diameter (Mode)[nm]*	D99.8 [nm]	Largest Particle Diameter, Upper bound (D100)[nm]
**E 171-a Fed**				
0.01 mg/ml, T=0	54	76	566	649
0.01 mg/ml, T=30	54	63	539	652
0.1 mg/ml, T=0	39	106	556	669
0.1 mg/ml, T=30	43	182	564	673
0.7 mg/ml, T=0	40	227	628	718
0.7 mg/ml, T=30	42	245	602	666
**E 171-a Fasted**				
0.01 mg/ml, T=0	59	138	575	667
0.01 mg/ml, T=30	53	98	567	746
0.1 mg/ml, T=0	38	85	599	665
0.1 mg/ml, T=30	40	64	597	704
0.7 mg/ml, T=0	37	188	666	723
0.7 mg/ml, T=30	36	42	717	771
**Chocolate candies**				
0.15 mg/ml, T=0	32	69	642	739
0.15 mg/ml, T=30	32	81	620	703
**Cappuccino**				
0.54 mg/ml, T=0	48	234	613	768
0.54 mg/ml, T=30	49	180	564	645

* In the presence of multimodal distributions, the value with the highest occurrence frequency was used for the evaluation of the results.

References

1. Mech, A.; Rauscher, H.; Rasmussen, K.; Babick, F.; Hodoroaba, V.-D.; Ghanem, A.; Wohlleben, W.; Marvin, H.; Brüngel, R.; Friedrich, C.M.; et al. The NanoDefine Methods Manual., doi:10.2760/02910.
2. Verleysen, E.; Brassinne, F.; Van Steen, F.; Waegeneers, N.; Cheyns, K.; Machiels, R.; Mathioudaki, S.; Jimenez, I.O.; Ledecq, M.; Mast, J. Towards a Generic Protocol for Measuring the Constituent Particle Size Distribution of E171 in Food by Electron Microscopy. *Food Control* **2022**, *132*, doi:10.1016/j.foodcont.2021.108492.
